# M-Current Expands the Range of Gamma Frequency Inputs to Which a Neuronal Target Entrains

**DOI:** 10.1186/s13408-018-0068-6

**Published:** 2018-12-05

**Authors:** Yujia Zhou, Theodore Vo, Horacio G. Rotstein, Michelle M. McCarthy, Nancy Kopell

**Affiliations:** 1 Department of Mathematics and Statistics, Boston University, Boston, USA; 2 Department of Mathematics, Florida State University, Tallahassee, USA; 3 Department of Biological Sciences, New Jersey Institute of Technology, Newark, USA

**Keywords:** Phase-amplitude coupling, Theta rhythm, Geometric singular perturbation theory, Averaging, Bistability, Multiple timescales, Biophysical modeling

## Abstract

Theta (4–8 Hz) and gamma (30–80 Hz) rhythms in the brain are commonly associated with memory and learning (Kahana in J Neurosci 26:1669–1672, 2006; Quilichini et al. in J Neurosci 30:11128–11142, 2010). The precision of co-firing between neurons and incoming inputs is critical in these cognitive functions. We consider an inhibitory neuron model with M-current under forcing from gamma pulses and a sinusoidal current of theta frequency. The M-current has a long time constant (∼90 ms) and it has been shown to generate resonance at theta frequencies (Hutcheon and Yarom in Trends Neurosci 23:216–222, 2000; Hu et al. in J Physiol 545:783–805, 2002). We have found that this slow M-current contributes to the precise co-firing between the network and fast gamma pulses in the presence of a slow sinusoidal forcing. The M-current expands the phase-locking frequency range of the network, counteracts the slow theta forcing, and admits bistability in some parameter range. The effects of the M-current balancing the theta forcing are reduced if the sinusoidal current is faster than the theta frequency band. We characterize the dynamical mechanisms underlying the role of the M-current in enabling a network to be entrained to gamma frequency inputs using averaging methods, geometric singular perturbation theory, and bifurcation analysis.

## Introduction

Gamma (30–80 Hz) and theta (4–8 Hz) oscillations are prominent rhythms observed in local field potentials recorded from entorhinal–hippocampal circuits [2, 5, 6]. Gamma oscillations often emerge at specific phases of the slower theta oscillations, a phenomenon known as theta–gamma cross-frequency coupling (CFC) [7, 8]. Entorhinal–hippocampal theta–gamma CFC is thought to be critical in the process of memory formation [9–12]. Computational modeling suggests theta–gamma CFC allows gamma oscillations from different regions to be coordinated temporally, since theta oscillations can be synchronized over larger spatial areas than gamma [13]. Precise coordination of spikes is critical for spike-time-dependent plasticity (STDP) thought to underlie mechanisms of learning and memory [14]. Models also suggest the theta component of the theta–gamma coupling may serve either as a phase reference for the alignment of the gamma oscillations [13, 15] or as a mechanism that periodically breaks communication between neuronal ensembles and allows the system to reset [16].

Here, using computational models, we find an additional, nonintuitive function for theta–gamma CFC: theta–gamma CFC can effectively allow one gamma oscillator to precisely regulate the gamma spiking of the theta–gamma targeted oscillator. We show that the underlying mechanism involves the theta timescale of an intrinsic membrane potassium current known as the M-current. This is surprising because the M-current (internal theta timescale) interacts with both external gamma and theta timescales. Even though gamma-aminobutyric acid (GABA) synaptic currents are a key ingredient in the generation of gamma oscillations [17], their presence is not enough to secure entrainment by a gamma input in the presence of a theta input in our model. Note that, in this paper, we emphasize the order of spike arrival by using the expression “a cell follows the inputs.” In particular, we only consider the type of entrainment where the output spikes occur after the input spikes, due to its implication in STDP [14].

For this purpose, we consider a small neuronal network consisting of a single cell with an inhibitory autapse, where the cell is provided with an M-current. Such a network can represent the simplest form of a pyramidal-interneuronal network, or PING [18]; the inhibitory autapse then represents the inhibitory feedback to the pyramidal cell. The M-current can exist in either excitatory cells [4] or inhibitory cells [19] in the network. Such a network exists in abundance in the brain [20–22], in particular in the hippocampus [23, 24]. In the latter case, it is known that there are both gamma [5] and theta [6, 25] inputs, as we use in our model. Thus, our model can represent hippocampal networks and their inputs, and possibly be generalized to other situations in which the target network receives gamma and theta inputs.

Mechanistically, the M-current plays a dual role in the network. In the subthreshold regime, it interacts with the external theta current, providing homeostasis and stabilizing the subthreshold voltage fluctuations in a frequency-dependent manner. In the spiking regime, it interacts with the external gamma pulses. It expands the range of frequencies over which the oscillator can follow. In particular, the M-current allows the oscillator to $1:1$ phase-lock to a rhythm slower than its natural frequency. Moreover, it enables the oscillator to be sparsely entrained by fast inputs (i.e., the oscillator skips some of the input cycles but always closely follows the inputs).

The M-current is able to provide subthreshold homeostasis as long as the external current is in the theta frequency band or slower. We show this by simulating an averaged system and observe that the homeostatic effect gradually wears off as the frequency of external current increases, which results in the loss of entrainment. The mechanism of M-current in the spiking regime is more complex. We first examine the phase plane of a reduced model to show that when the oscillator with M-current receives pulses, the reset point is farther away from the knee of the voltage nullcline (in a vicinity of the slow manifold created by the M-current) than when there is no pulse. This is due to pulse-triggered spikes having a larger amplitude and therefore generating a bigger amount of M-current than autonomous spikes. Hence, after an external pulse triggers a spike, it takes the oscillator longer than its natural interspike interval to generate the next spike, allowing the cell to be able to follow a rhythm slower than its natural frequency. Without the M-current, there is no slow manifold; the interspike intervals are the same with autonomous spikes and triggered spikes. Next, utilizing geometric singular perturbation theory, we study a network of an input cell (E-cell) forcing an oscillator with an inhibitory autapse (I-cell). Considering dynamics in the singular limit of the slow timescale, two relevant fold structures exist: the E-fold determines the spiking of the E-cell and the I-fold determines the spiking of the I-cell. Our results show that the presence of an M-current alters the position of the I-fold such that the singular orbit of limit cycle always reaches the E-fold first, allowing the I-cell to be driven precisely (i.e., sparsely entrained) by the E-cell (external pulses). While the reduced model tells us that the interspike interval is lengthened due to the position of reset point and the slow manifold created by the M-current, the geometric singular perturbation analysis tells us that the fold is also further away from the trajectory when the M-current is stronger.

In addition, we present a special case, where the oscillator with M-current can be entrained by an arbitrarily slow rhythm, due to bistability. We also discuss the similarities and differences between an M-current and an h-current in Sect. 6, as h-current is also a resonant current and it operates at a similar timescale as the M-current [4].

## Mathematical Model

**Fig. 1:**
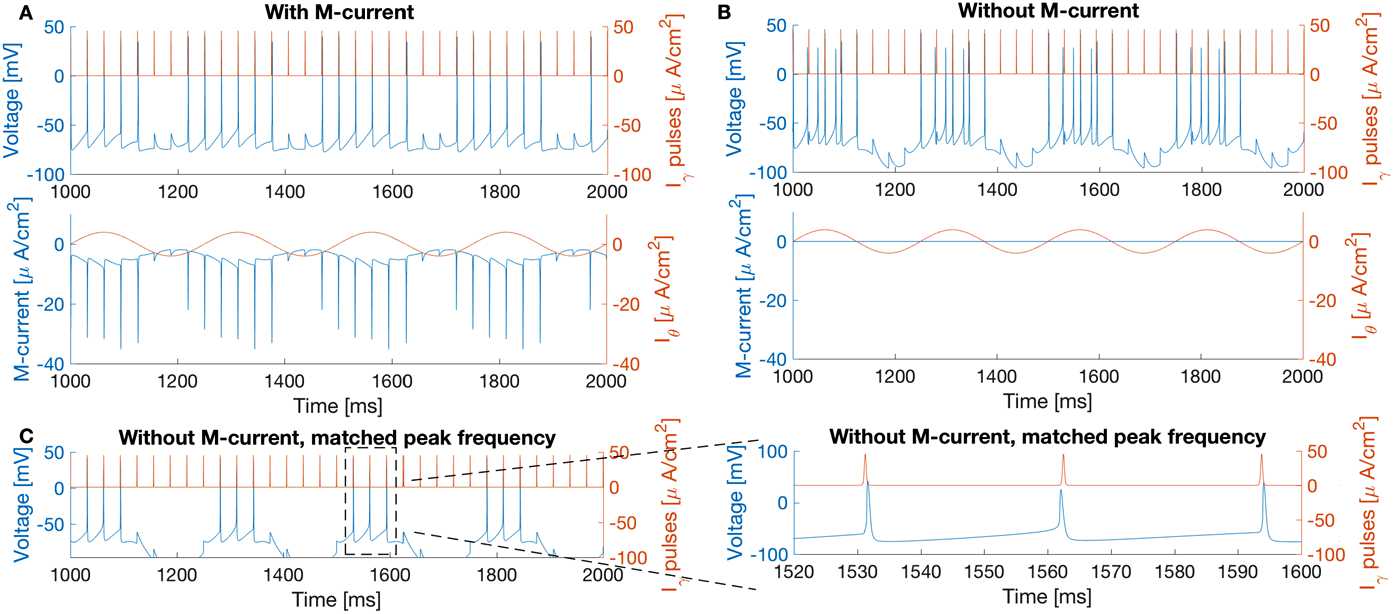
Comparison between simulations of (1) with the M-current (panel **A**, $g_{M}=1.5$ mS/cm^2^, $I_{\mathit {ton}}=5$ *μ*A/cm^2^) and without the M-current (panel **B**, $g_{M}=0$ mS/cm^2^, $I_{\mathit {ton}}=0.55$ *μ*A/cm^2^), adjusted to have the same natural frequency (16 Hz). Top and bottom rows: Voltage trace of the I-cell is in blue. External gamma pulses (32 Hz) are in red. Middle row: M-current is in blue. Theta forcing is in red. In **A** (with the M-current), the spikes of the I-cell align with the external pulses. In **B**, the I-cell fails to follow the same pulses when the M-current is absent. The voltage envelope of the I-cell with M-current also has much less variation than the one without M-current. **C**: Simulation of (1) without M-current, but with the same excitability (34 Hz) at the peak of theta forcing as in **A** ($g_{M}=0$ mS/cm^2^, $I_{\mathit {ton}}=-1.7$ *μ*A/cm^2^). One of the I-cell spikes during each theta cycle comes earlier than the external pulse

The objective is to study how an M-current in a small network improves the precision of co-firing between gamma inputs and the target network in the presence of a current of theta frequency. We consider a Hodgkin–Huxley-like model for a cell with an autapse and the M-current (referred to as the I-cell in the rest of the paper), which can also be thought as a PING network (by regarding the cell as the pyramidal cell in PING and the inhibitory autapse as the inhibitory feedback from interneurons to pyramidal cells). Theta input is modeled as a slowly changing (sinusoidal) oscillation (4 Hz) representing incoming activity from a large ensemble of neurons. In contrast, we hypothesize that gamma inputs are more synchronous, needed to mark the precise moment at which they arrive. To this end, we make the gamma input very sharp over a short time frame (less than 1 ms) to resemble excitatory input from spike trains (see red trace in the top panel of Fig. 1).

The differential equations describing the neuron are as follows:

1 $$\begin{aligned} C\frac{dV}{dt}&=I_{L}(V)+I_{K}(V,n)+I_{\mathit {Na}}(V,h)+I_{\mathit {GABA}_{A}}(V,s)+I_{M}(V,w)+I(t), \\ \frac{dn}{dt}&=\frac{n_{\infty}(V)-n}{\tau_{n}(V)}=\phi_{n}(V,n), \\ \frac{dh}{dt}&=\frac{h_{\infty}(V)-h}{\tau_{h}(V)}=\phi_{h}(V,h), \\ \frac{ds}{dt}&=\frac{1}{2} \biggl[ 1+\tanh \biggl( \frac{V}{4} \biggr) \biggr] \frac{1-s}{\tau_{r}}-\frac{s}{\tau_{d}}=\phi_{s}(V,s), \\ \frac{dw}{dt}&=\frac{w_{\infty}(V)-w}{\tau_{M}(V)}=\phi_{w}(V,w), \end{aligned}$$

where $I_{L}$, $I_{K}$, $I_{\mathit {Na}}$, $I_{\mathit {GABA}_{A}}$, and $I_{M}$ are the leak current, potassium current, sodium current, GABA_*A*_ current (inhibitory autapse), and M-current (a slow, noninactivating potassium current), respectively. They take the form

2 $$ \begin{aligned} I_{L}(V)&=g_{L}(E_{L}-V), \\ I_{K}(V,n)&=g_{K}n^{4}(E_{K}-V), \\ I_{\mathit {Na}}(V,h)&=g_{\mathit {Na}}m^{3}_{\infty}(V)h(E_{\mathit {Na}}-V), \\ I_{\mathit {GABA}_{A}}(V,s)&=g_{s} s(E_{s}-V), \\ I_{M}(V,w)&=g_{M}w(E_{M}-V). \end{aligned} $$

The external forcing term, $I(t)$, will be described later. Parameter values and units are listed in Table A.1 in Appendix 1. Details of the steady state activation/inactivation functions and time constant functions ($m_{\infty}$, $n_{\infty}$, $h_{\infty}$, $w_{\infty}$, $\tau _{n}$, $\tau_{h}$, $\tau_{M}$) are provided in Table A.2 in Appendix 1.

**Table A.1:** Baseline parameter values for (1) and (7)

Conductance (mS/cm^2^)		Reversal potential (mV)		Time constant (ms)		Capacitance (*μ*F/cm^2^)	
$g_{L}$	0.1	$E_{L}$	−65	$\tau_{r}$	0.3	*C*	1
$g_{K}$	9	$E_{K}$	−90	$\tau_{d}$	9		
$g_{\mathit {Na}}$	35	$E_{\mathit {Na}}$	55				
$g_{s}$	1	$E_{s}$	−80				
$g_{M}$	1.5	$E_{M}$	−90

**Table A.2:** Expressions for infinity and *τ* functions in Eq. (1)

$m_{\infty}(V)=\frac{\alpha_{m}(V)}{\alpha_{m}(V)+\beta_{m}(V)}$	$\alpha_{m}(V)=\frac{0.1(V+35)}{1-e^{-\frac{V+35}{10}}}$	$\beta _{m}(V)=4e^{-\frac{V+60}{18}}$
$n_{\infty}(V)=\frac{\alpha_{n}(V)}{\alpha_{n}(V)+\beta_{n}(V)}$	$\alpha_{n}(V)=\frac{-0.01(V+34)}{e^{-0.1(V+34)}-1}$	$\beta _{n}(V)=0.125e^{-\frac{V+44}{80}}$
$h_{\infty}(V)=\frac{\alpha_{h}(V)}{\alpha_{h}(V)+\beta_{h}(V)}$	$\alpha_{h}(V)=0.07e^{-\frac{V+58}{20}}$	$\beta_{h}(V)=\frac {1}{e^{-0.1(V+28)}+1}$
$w_{\infty}(V)=\frac{1}{1+e^{-\frac{V+35}{10}}}$	$\tau_{M}(V)=\frac {400}{3.3e^{\frac{v+35}{20}}+e^{-\frac{v+35}{20}}}$	*ϕ* = 5
$\tau_{n}(V)=\frac{1}{\alpha_{n}(V)+\beta_{n}(V)}\cdot\frac{1}{\phi }$	$\tau_{h}(V)=\frac{1}{\alpha_{h}(V)+\beta_{h}(V)}\cdot\frac {1}{\phi}$	

The system (1) features at least two intrinsic timescales. The GABA_*A*_ autapse has a decay time constant of $\tau_{d}=9$ ms. Previous works showed that the GABA_*A*_ synapse is a key ingredient in gamma oscillations [26, 27]. The M-current is proposed in [4] to contribute to a theta rhythm. The time constant of the M-current is voltage dependent and is ten times as long (∼90 ms) as the GABA_*A*_ decay time near the spiking threshold (around −60 mV).

The cell is receiving external inputs at gamma and theta frequencies. The forcing term $I(t)$ is

3 $$ I(t)=I_{\mathit {ton}}+aI_{\gamma}(t)+bI_{\theta}(t), $$

where $I_{\mathit {ton}}$ is a tonic current, $I_{\gamma}$ is a purely excitatory pulsatile input which represents the gamma forcing, and $I_{\theta}$ is a slowly varying sinusoidal current with zero average that represents the theta forcing. $I_{\mathit {ton}}$ sets the natural spiking frequency of the I-cell, that is, the spiking frequency of the I-cell without any external theta or gamma forcing. The parameters *a* and *b* control the strengths of the gamma and theta forcing. The specific functions and parameters that describe the forcing terms are given in Table A.3 in Appendix 1. At the baseline parameter values, the gamma forcing frequency is 32 Hz and the theta forcing frequency is 4 Hz.

**Table A.3:** Functions and parameters that define the forcing term (3)

$I_{\gamma}(t)=C_{\gamma}\cdot J_{\gamma}(t)$	$I_{\theta}(t)=\sin (\frac{2\pi}{T_{\theta}}t)$	$I_{\mathit {ton}}=5$ *μ*A/cm^2^
$J_{\gamma}(t)=e^{\alpha_{\gamma}[\cos(\frac{\pi t}{T_{\gamma }})]^{1024}}-1$	$T_{\theta}=250$ ms	*a* = 0.6
$C_{\gamma}=\frac{1}{\frac{1}{T_{\gamma}}\int_{0}^{T_{\gamma }}J_{\gamma}(t)\,dt}$	$\alpha_{\gamma}=5$	*b* = 4
$T_{\gamma}=31.25$ ms		

## M-Current Allows the I-Cell to Follow Gamma Input in the Presence of a Slow Theta Forcing

Simulation results are presented in this section. We will provide some mathematical analysis of these results in Sects. 4 and 5. Simulations of (1), subject to (3), show that, in this particular model, the M-current promotes the oscillator’s entrainment to gamma pulses. In Fig. 1, we consider the response of the I-cell when the M-current is present (Fig. 1A) and absent (Fig. 1B). We lowered $I_{\mathit {ton}}$ in the I-cell without M-current such that the natural spiking rate (measured without gamma or theta forcing) is 16 Hz in both cases. Note that, since the theta forcing is slowly changing (in comparison with gamma pulses), we can think of it as a slowly changing tonic current. Then 16 Hz can also be thought of as the natural frequency when the theta forcing is at 0. The most distinctive difference between the two regimes is that the one with M-current is able to follow the pulses but the one without M-current cannot.

In addition to the differences in entrainment, we also find that the voltage envelope of the model with M-current is almost flat, while the voltage envelope of the model without M-current fluctuates with the theta forcing (Fig. 1). This is consistent with the fact that the M-current acts as a homeostatic current with respect to voltage [4]. Here we define homeostasis as the ability of an intrinsic current to counteract subthreshold fluctuations in membrane excitability that come from an external oscillatory forcing.

**Fig. 2:**
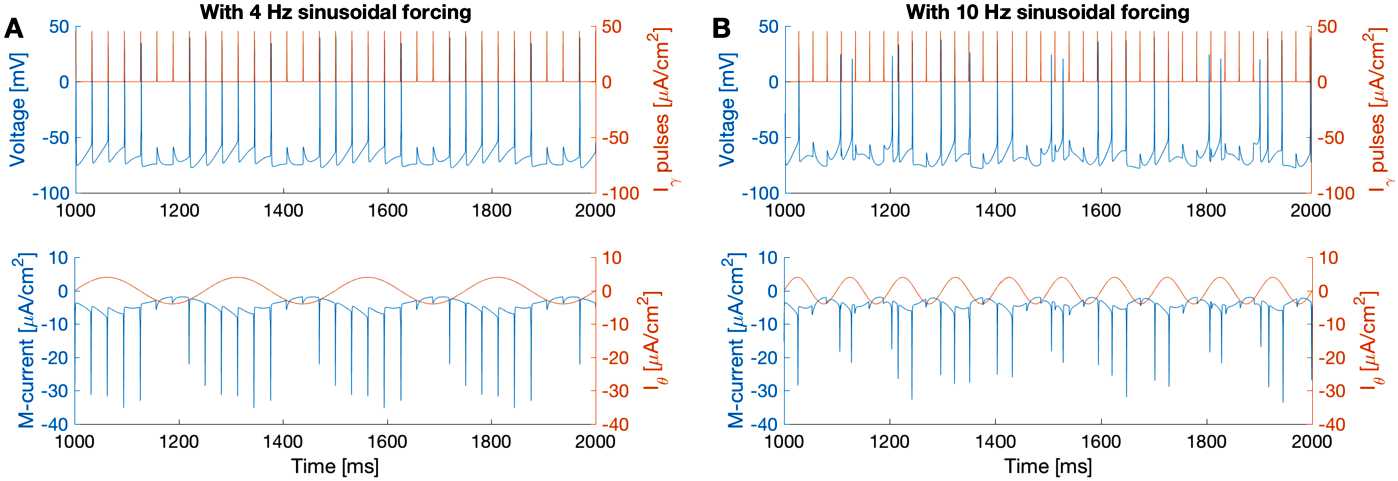
The I-cell loses its ability to phase-lock to gamma pulses when the sinusoidal forcing frequency increases from 4 Hz (**A**) to 10 Hz (**B**). Simulated from (1) with $T_{\theta }=250$ ms (**A**) and $T_{\theta}=100$ ms (**B**). Color scheme is as in Fig. 1

Interestingly, the homeostatic effect is reduced if the sinusoidal forcing is too fast. Here we simulate (1) with two different values of $T_{\theta}$ (theta forcing period; see Table A.3). In Fig. 2A, the I-cell receives 32 Hz gamma pulses and a 4 Hz sinusoidal forcing ($T_{\theta }=250$ ms), the same as in Fig. 1A. In Fig. 2B, with a 10 Hz sinusoidal forcing ($T_{\theta }=100$ ms), the I-cell does not follow gamma pulses; the voltage also fluctuates more than with 4 Hz sinusoidal forcing.

However, the homeostasis due to the M-current is not the only reason that the I-cell with M-current can follow gamma pulses. Although the natural frequency of the I-cells with and without M-current are both 16 Hz (in the absence of theta forcing), the I-cell without M-current becomes more excitable at the peak of theta than the I-cell with M-current, since it does not have the M-current to oppose the rise of theta forcing. In Fig. 1C, the tonic input is adjusted such that, at the peak of theta, the cell without M-current has the same natural frequency as the cell with M-current (34 Hz, measured without rhythmic inputs but with a constant current at $I(t)=I_{\mathit {ton}}+b$). As shown in the blow-up in Fig. 1C, during each theta cycle, one spike of the I-cell without M-current precedes the forcing. By contrast, the I-cell with M-current always spikes after the pulses. This change in spike arrival order has important implications in plasticity [14], since potentiating versus depressing synaptic strengths depends on whether the input spike arrives before or after the target spike (see Discussion). It is important to note that the frequency of gamma input (32 Hz) is slower than the natural frequency of the I-cell at the peak of theta forcing (34 Hz). Although it is somewhat intuitive why a faster gamma input could force a slower gamma oscillator, it is not at all obvious how, with M-current, a slower (32 Hz) forcing input can pace a faster oscillator.

**Fig. 3:**
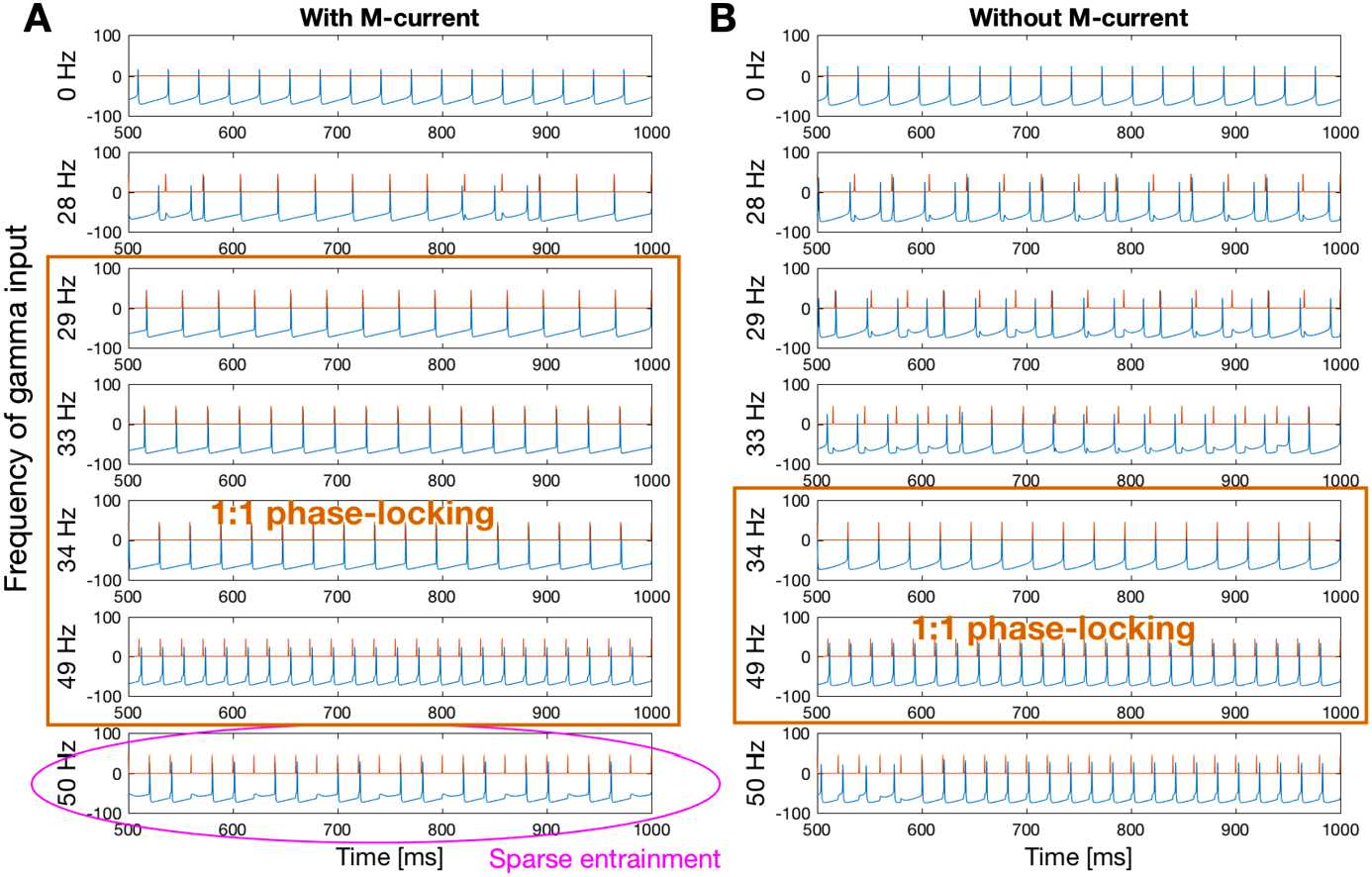
The I-cell with M-current has a larger $1:1$ phase-locking region (**A**, 29 Hz to 49 Hz) than the I-cell without M-current (**B**, 34 Hz to 49 Hz). The I-cell with M-current can phase-lock to pulses slower than its natural frequency (34 Hz). With pulses faster than the $1:1$ phase-locking band, the spikes of the I-cell with M-current always align with incoming pulses (sparse entrainment). Without the M-current, the I-cell has phase lag when being forced by faster pulses. Voltage traces of the I-cell are in blue. External pulses are in red. External pulse frequencies are labeled on the left

We find that the presence of the M-current significantly expands the frequency range where $1:1$ phase-locking occurs. In Fig. 3, we compare two I-cells of natural frequency 34 Hz, with and without M-current, forced by external pulses of frequencies both above and below 34 Hz (but without sinusoidal forcing). The lower bound of the $1:1$ phase-locking region of the cell without M-current coincides with the cell’s natural frequency (34 Hz). However, the lower bound of the $1:1$ phase-locking region of the cell with M-current is 29 Hz, slower than the cell’s natural frequency. The upper bound of the $1:1$ phase-locking region (49 Hz) is the same for both cells, not affected by the M-current. When the external pulses are faster than 49 Hz (last row of Fig. 3), the I-cell with M-current can be sparsely entrained: it spikes in alignment with some incoming pulses but skips others. Without the M-current, the I-cell develops a slowly increasing phase lag when being forced by pulses faster than the $1 : 1$ phase-locking range.

In summary, the M-current has two roles in allowing the I-cell to follow gamma pulses in the presence of theta forcing. One is the subthreshold homeostatic effect of the M-current, which we will explore in the next section, using averaging theory. The other is the interaction between the M-current and external pulses in the spiking regime. The M-current enables the oscillator to phase-lock to a rhythm slower than the oscillator’s natural frequency, and be sparsely entrained by fast inputs. We investigate the reasons for this analytically in Sect. 5 using a reduced model, geometric singular perturbation theory, and bifurcation analysis.

## Subthreshold Homeostasis: The Ability of the M-Current to Stabilize Voltage Is Gradually Reduced as the Sinusoidal Forcing Frequency Increases

Figure 2 shows that the homeostatic effect of the M-current can be sustained with 4 Hz sinusoidal forcing but is reduced with 10 Hz sinusoidal forcing (Fig. 2). To get a quantitative understanding of how the frequency of the forcing influences the homeostasis, we will use averaging theory to average over the output spikes.

It has been shown that the M-current provides the necessary negative feedback effect to create (subthreshold) membrane potential resonance in response to theta inputs [3, 4]. This effect may extend to the spiking regime, but it cannot be captured in a straightforward way using available methods measuring subthreshold resonance. Moreover, the M-current is built up in the spiking regime. Here we use the averaging method [28] to define a new measure to quantify the subthreshold fluctuations of the voltage and gating variables when the neuron is spiking.

To set up the averaged system, we need to identify the fast and slow variables in the system. In (1), the voltage *V* is the fast variable. All other variables are slow. (Details of the timescale separation can be found in Sect. 5.2.1.) The averaged equations are

4 $$\begin{aligned} \begin{aligned} C\frac{dV}{dt}&=I_{L}(V)+I_{K}(V, \bar{n})+I_{\mathit {Na}}(V,\bar{h})+I_{\mathit {GABA}_{A}}(V,\bar{s})+I_{M}(V, \bar{w})+I_{\mathit {ton}} + bI_{\theta}, \\ \frac{d \bar{x}}{dt}&= \textstyle\begin{cases} \phi_{x}(v_{\mathit {rest}},\bar{x}), \\ \quad \text{if $V$ goes to a fixed point when $\bar{n}$, $\bar{h}$, $\bar{s}$, $\bar{w}$ are frozen}, \\ \frac{1}{T(\bar{n}, \bar{h}, \bar{s},\bar{w})} \int_{0}^{T(\bar{n}, \bar{h}, \bar{s},\bar{w})} \phi_{x}(v_{\mathit {spike}}(\bar{n}, \bar{h}, \bar{s},\bar{w},t),\bar{x}) \,dt, \\ \quad \text{if $V$ converges to a limit cycle when $\bar{n}$, $\bar{h}$, $\bar{s}$, $\bar{w}$ are frozen}, \end{cases}\displaystyle \end{aligned} \end{aligned}$$

where $x=n,h,s,w$, $v_{\mathit {rest}}$ is the value of *V* at the fixed point, $T(\bar{n}, \bar{h}, \bar{s},\bar{w})$ is the period of the limit cycle, and $v_{\mathit {spike}}(\bar{n}, \bar{h}, \bar{s},\bar{w},t)$ is the voltage trace during one period of the limit cycle, and the overbar indicates an averaged variable. All functions, expressions of currents, and parameter values are the same as in (1). Note that there are no gamma pulses in (4), because we want to measure the voltage fluctuations in the spiking regime (which are brought about by the sinusoidal current), without the influence of external pulses.

**Fig. 4:**
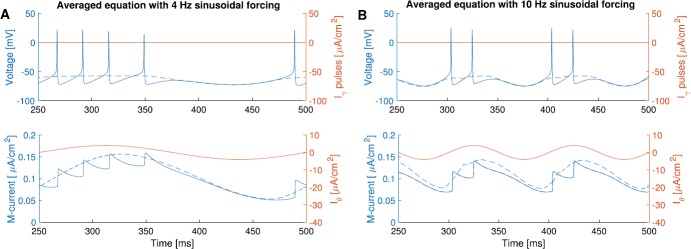
Simulations of the averaged system (4) (dashed curves), with 4 Hz (**A**, $T_{\theta}=250$ ms) and 10 Hz (**B**, $T_{\theta}=100$ ms) sinusoidal forcing, superimposed on simulation of (1) without gamma pulses ($a=0$, solid curves). The solution of the averaged equation tracks the solution of the original system very well. The color scheme is the same as in Fig. 1

Figure 4 shows that the solution of the averaged system (4) closely approximates the fluctuations of voltage and the M-current gating variable *w*, under sinusoidal forcing of different frequencies (4 Hz and 10 Hz). Thus, we use the solution of the averaged system (4) as a measure of the overall change of the variables in (1).

**Fig. 5:**
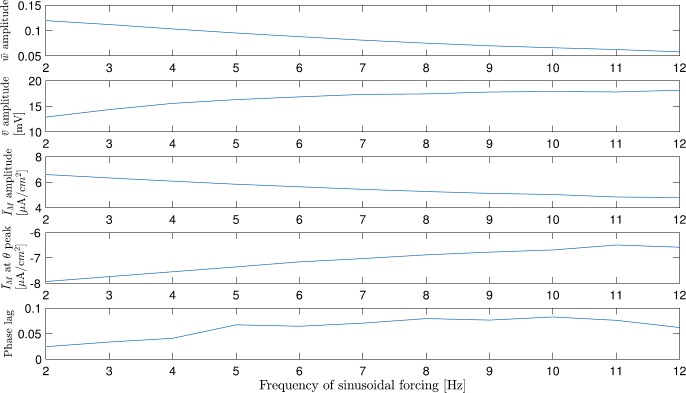
The amplitudes of the *w̄*, *v̄*, $\bar{I}_{M}$, the amount of $\bar{I}_{M}$ at $I_{\theta}$ peak, and the phase lag between $\bar{I}_{M}$ and $I_{\theta}$ change as the frequency of the sinusoidal forcing increases. The amplitude of *v̄* and the phase lag increase, while the *w̄* amplitude, $\bar{I}_{M}$ amplitude, and the absolute amount of $\bar{I}_{M}$ at $I_{\theta}$ peak decrease

The solution of the averaged system (4) reveals a few things that are not obvious from the solution of the original system (1). We observe the following trends as the frequency of the sinusoidal forcing increases (Fig. 5):

- The amplitude of the averaged M-current gating variable *w̄* decreases.
- The averaged voltage is less constant and affected more by the sinusoidal forcing.
- The amplitude of the averaged M-current ($\bar{I}_{M}=g_{M} \bar{w}(E_{M}-V)$) decreases.
- The amount (absolute value) of the averaged M-current at the peak of the sinusoidal forcing decreases.
- The phase lag (in percentage of the sinusoidal forcing cycle) between the averaged M-current peak and the sinusoidal forcing peak increases.

These results confirms that, in the spiking regime, the M-current cannot keep up with the change in the sinusoidal forcing if the latter is too fast. The homeostatic effect is reduced gradually, as the sinusoidal forcing frequency increases, until it is not enough to keep the cell from spiking before a pulse arrives. The functional consequence of this is that with faster sinusoidal forcing, there is not enough M-current to counteract the amount of excitation at the peak of the sinusoidal forcing.

## Spiking Regime: Interaction Between M-Current and External Pulses

Besides homeostasis, the other function of the M-current that contributes to the entrainment we observed in Fig. 1 (Sect. 3) is the expansion of the frequency range that a cell with M-current can follow. The M-current extends both the lower and the upper limits of the frequency range over which the cell can be entrained. In this section, we explore some mathematical structures that offer insight to the expansion of the entrainment frequency range. First, we use a reduced model to show that having an M-current creates a slow manifold. This model is two-dimensional in the subthreshold regime. The reset point is higher from the knee of the *V*-nullcline when there is an external pulse than when the cell is firing autonomously. Thus, the interspike interval is effectively lengthened, allowing the cell to be phase-locked to a rhythm slower than its natural frequency. Second, utilizing geometric singular perturbation theory, we provide a visualization of the fold structure positions, suggesting that the presence of M-current moves one of the folds such that when the I-cell fires, its spike always occur after the external pulse (sparse entrainment). Third, we present a special case where, in a certain parameter regime, the I-cell with M-current can phase-lock to an arbitrarily slow rhythm due to bistability.

### M-Current Introduces a Slow Manifold and External Pulses Lengthen the Interspike Interval

To capture the essence of the M-current’s role in the system’s dynamics, we analyze a 2D reduced model with artificial spikes. We will show that the M-current introduces a slow manifold and the external pulse changes the reset point position on the slow manifold, enabling the cell with M-current to phase-lock to inputs slower than its natural frequency.

The reduced (2D) model approximates the dynamics of the I-cell in the subthreshold regime and the onset of spikes. Spikes are added artificially after the voltage leaves the subthreshold regime. This is indicated by a threshold value. The spike width has been set to 1 ms and the spike height varies according to whether the gamma input is present or not to reflect our observations using the full model (see Fig. 1). The reduced model includes the leak current, the M-current and the sodium current where both the activation and the inactivation gating variables are slaved to voltage. The equations describing the subthreshold dynamics ($V<-40$ mV) are as follows.

5 $$ \begin{aligned} C\frac{dV}{dt}&=I_{L}(V)+I_{\mathit {NaT}}(V)+I_{M}(V,w)+I_{\mathit {app}}, \\ \frac{dw}{dt}&=\frac{w_{\infty}(V)-w}{\tau_{M}(V)}=\phi_{w}(V,w), \end{aligned} $$

where

6 $$ \begin{aligned} I_{\mathit {NaT}}(V)&=g_{\mathit {Na}}m^{3}_{\infty}(V)h_{\infty}(V) (E_{\mathit {Na}}-V), \end{aligned} $$

and $I_{\mathit {app}}$ is a constant. Other functions and parameters are the same as in (1). When *V* reaches −40 mV, an artificial spike is inserted and the voltage is reset to −64 mV. We make the spike height bigger for a spike triggered by an external pulse than for an autonomous spike as in Fig. 1B. This is essential in reproducing Fig. 1 using the reduced model. We eliminate the autapse to highlight the role of the M-current; we can qualitatively reproduce the results of Figs. 1 and 3 without an autapse. We also eliminate the potassium current and replace $I_{\mathit {Na}}$ by $I_{\mathit {NaT}}$ because, in the subthreshold regime, the potassium current is close to zero and the inactivating variable (*h*) of the transient sodium current is close to its steady state. This change erases the spiking mechanism (except for spike onset) while preserving the subthreshold dynamics.

**Fig. 6:**
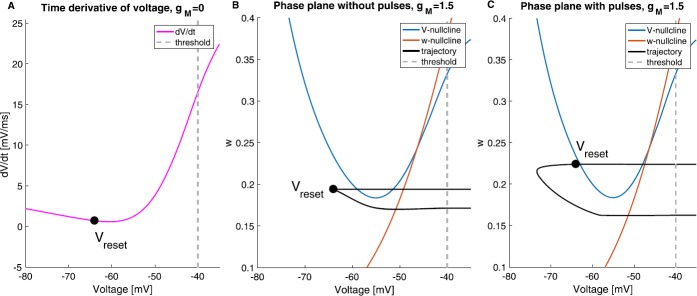
The graph of derivative and phase planes of (5) with 3 sets of parameters. **A**: The time derivative of voltage is always positive and the cell without M-current has a natural frequency of 34 Hz ($g_{M}=0$ mS/cm^2^, $I_{\mathit {app}}=0.68$ *μ*A/cm^2^, $a=0$). **B**: The phase plane of the cell with M-current. The cell is firing at 34 Hz without external inputs ($g_{M}=1.5$ mS/cm^2^, $I_{\mathit {app}}=9.1$ *μ*A/cm^2^, $a=0$). **C**: The same cell as in **B** but with 32 Hz external pulses. The voltage resets to a higher point near the *V*-nullcline than in **B** ($g_{M}=1.5$ mS/cm^2^, $I_{\mathit {app}}=9.1$ *μ*A/cm^2^, $a=0.6$)

Without M-current ($g_{M}=0$), (5) would be one-dimensional (of quadratic integrate-and-fire type). The derivative of voltage is plotted in Fig. 6A. After a spike has occurred, whether it is autonomous or triggered by an input, the voltage is reset to $V_{\mathit {reset}}$ (black dot). Then the voltage will keep increasing (since the derivative is positive) until it reaches the threshold and then produces a spike. The time that the voltage takes to rise from $V_{\mathit {reset}}$ to threshold is the cell’s interspike interval. If the incoming pulses are slower than the natural frequency, i.e. the time between to consecutive pulses is longer than the cell’s interspike interval, the cell will fire before the pulse arrives because the voltage has reached the threshold. Therefore, entrainment is not possible.

Having the M-current in the system introduces a slow manifold (a vicinity of the *V*-nullcline in Figs. 6B and C). The trajectory travels close to the slow manifold until it reaches the knee. When there is no external pulse (Fig. 6B), the trajectory returns to the reset point (black dot) after a spike. When the spike is triggered by a pulse, however, the reset point is higher up, which is a result of a bigger spike size. If there is no next pulse, it would take the trajectory longer to arrive at the knee in Fig. 6C (triggered spike) than in Fig. 6B (autonomous spike). With M-current, the effective interspike interval when there are external pulses is longer than the natural interspike interval, because of the higher reset point. Hence, the cell with M-current can phase-lock to rhythms slower than its natural frequency.

The advantage of using the reduced model is its low dimensionality, which allows us to easily draw and study its phase plane. One limitation of the reduced model is its inability to capture the difference in spike sizes in the presence and absence of external gamma inputs. More specifically, the adjustments are inferred from the simulations of the full model. Thus, the subthreshold dynamics alone do not capture the effect of external pulses on the system. The difference in spike size is crucial; it leads to M-current building up to a higher value during a triggered spike than an autonomous spike. Next, we study a network model that takes external pulses into account to help us understand sparse entrainment.

### M-Current Moves the Fold Structure to Enable Sparse Entrainment

To understand how having the M-current leads to sparse entrainment when the input frequency is greater than the $1:1$ phase-locking frequency range, we utilize geometric singular perturbation analysis on a network model. First, we formally define the timescales and the associated subsystems, laying the groundwork for our analysis. Then we identify special geometric structures in our model called folds, which correspond to firing thresholds. Next, with some projections, we visualize the fold in a 3D space. We also explain their critical role in phase-locking. Last, the visualizations suggest that M-current alters the position of the I-cell firing threshold such that the trajectory of the network always arrives at the E-cell firing threshold before the I-cell firing threshold, resulting in entrainment when the input frequency is in an appropriate range and sparse entrainment when the input frequency is above that range.

#### Geometric Singular Perturbation Analysis

To build a network model, we recast the non-autonomous problem (1) subject to (3) as an autonomous problem by interpreting the gamma pulses as the output of an excitatory cell (E-cell). The E-cell is described by the equations:

7 $$\begin{aligned} C\frac{dV_{2}}{dt}&=I_{L}(V_{2})+I_{K}(n_{2},V_{2})+I_{\mathit {Na}}(h_{2}, V_{2})+J, \\ \frac{dn_{2}}{dt}&=\frac{n_{\infty}(V_{2})-n_{2}}{\tau_{n}(V_{2})}, \\ \frac{dh_{2}}{dt}&=\frac{h_{\infty}(V_{2})-h_{2}}{\tau_{h}(V_{2})}, \end{aligned}$$

where *J* is a constant current that controls the frequency of the E-cell (external pulses). The leak, potassium, sodium currents are as the same as in (2). The values of maximal conductances and reversal potentials are the same as in (1) and (2), which are provided in Table A.1. At the baseline value $J=0.5$ *μ*A/cm^2^, the E-cell is firing at 32 Hz, the same as $I_{\gamma}$ in (3) in Sect. 2. The E-cell is connected to the I-cell through a one-way excitatory synapse. The gating variable of the synapse is defined by

8 $$ \frac{ds_{e}}{dt}=\frac{1}{2} \biggl[ 1+\tanh \biggl( \frac{V_{2}}{4} \biggr) \biggr] \frac{1-s_{e}}{\tau_{r_{e}}}-\frac{s_{e}}{\tau _{d_{e}}}, $$

with $\tau_{r_{e}}=0.1$ ms and $\tau_{d_{e}}=0.75$ ms. With these modifications, we replace the gamma forcing term $aI_{\gamma}(t)$ in (3) by

9 $$ g_{f}s_{e}(E_{e}-V), $$

where $g_{f}=0.5$ mS/cm^2^ and $E_{e}=0$ mV. Thus, the system we now consider is (1), (7), and (8) subject to (3), where in (3), the gamma forcing term $a I_{\gamma}(t)$ is replaced by (9) and $b=0$. We point out that the subsystem (7) describing the E-cell is independent of (1), which is convenient in the analysis, as we will show later.

To uncover the timescales in the network model, we non-dimensionalize the system. We choose the reference scales and perform changes of variables as follows: $k_{v}=100$ mV, $g_{\mathit {ref}}=10$ mS/cm^2^, $k_{t}=\max_{-80\le V\le-50}(\tau_{M}(V))\approx108$ ms, $\bar{\tau }_{x}=\max_{-80\le V\le-50}(\tau_{x}(V))$ for $x=n,h$, $\bar{\tau }_{s}=\max(\tau_{r},\tau_{d})=9$ ms, $\bar{\tau}_{{s_{e}}}=\max (\tau_{r_{e}},\tau_{d_{e}})=0.75$ ms. We let $V=k_{v}v$, $V_{2}=k_{v}v_{2}$, $E_{x}=k_{v}e_{x}$ for $x=L,K,\mathit {Na},s,M,e$, $g_{x}=g_{\mathit {ref}}\bar{g}_{x}$ for $x=n,h,s,w$, $t=k_{t} \tau$. There is no need to rescale the gating variables (*n*, *h*, *s*, *w*, $n_{2}$, $h_{2}$, $s_{e}$), since they are unitless and vary between 0 and 1. The non-dimensionalized full (E-I) system is

10 $$\begin{aligned} \epsilon\frac{dv}{d\tau}&=\bar{g}_{L}(e_{L}-v)+ \bar{g}_{K}n^{4}(e_{K}-v)+\bar {g}_{\mathit {Na}}m^{3}_{\infty}(k_{v} v)h(e_{\mathit {Na}}-v)+\bar{g}_{s} s(e_{s}-v) \\ & \quad {} +\bar{g}_{M}w(e_{M}-v)+\bar{g}_{f}s_{e}(v_{e}-v)+ \frac{I_{\mathit {ton}}}{k_{v} g_{\mathit {ref}}}=f_{1}(v,n,h,s,w,s_{e};I_{\mathit {ton}}), \\ \frac{dn}{d\tau}&=k_{t}\frac{n_{\infty}(k_{v} v)-n}{\tau_{n}(k_{v} v)}=p_{1}(v,n), \\ \sigma_{1}\frac{dh}{d\tau}&=\frac{k_{t}}{\bar{\tau}_{n}} \frac{h_{\infty}(k_{v} v)-h}{\tau_{h}(k_{v} v)/\bar{\tau}_{h}}=p_{2}(v,h), \\ \sigma_{2}\frac{ds}{d\tau}&=\frac{k_{t}}{\bar{\tau}_{n}} \biggl( \frac{1}{2} \biggl[ 1+\tanh \biggl( \frac{k_{v} v}{4} \biggr) \biggr] \frac{1-s}{\tau_{r}/\bar{\tau}_{s}}-\frac{s}{\tau_{d}/\bar{\tau}_{s}} \biggr) =p_{3}(v,s), \\ \frac{dw}{d\tau}&=k_{t}\frac{w_{\infty}(k_{v} v)-w}{\tau_{M}(k_{v} v)}=g(v,w), \\ \epsilon\frac{dv_{2}}{d\tau}&=\bar{g}_{L}(e_{L}-v_{2})+ \bar{g}_{K}n_{2}^{4}(e_{K}-v_{2})+ \bar{g}_{\mathit {Na}}m^{3}_{\infty}(k_{v} v_{2})h_{2}(e_{\mathit {Na}}-v_{2}) \\ & \quad {} +\frac{J}{k_{v} g_{\mathit {ref}}}=f_{2}(v_{2},n_{2},h_{2};J), \\ \frac{dn_{2}}{d\tau}&=k_{t}\frac{n_{\infty}(k_{v} v_{2})-n_{2}}{\tau _{n}(k_{v} v_{2})}=p_{4}(v_{2},n_{2}), \\ \sigma_{1}\frac{dh_{2}}{d\tau}&=\frac{k_{t}}{\bar{\tau}_{n}} \frac{h_{\infty}(k_{v} v_{2})-h_{2}}{\tau_{h}(k_{v} v_{2})/\bar{\tau}_{h}}=p_{5}(v_{2},h_{2}), \\ \sigma_{3}\frac{ds_{e}}{d\tau}&=\frac{k_{t}}{\bar{\tau}_{n}} \biggl( \frac{1}{2} \biggl[ 1+\tanh \biggl( \frac{k_{v} v_{2}}{4} \biggr) \biggr] \frac{1-s_{e}}{\tau_{r_{e}}/\bar{\tau}_{se}}-\frac{s_{e}}{\tau _{d_{e}}/\bar{\tau}_{se}} \biggr) =p_{6}(v_{2},s_{e}), \end{aligned}$$

where $\epsilon=\frac{C}{g_{\mathit {ref}} k_{t}}\approx0.0009 \ll1$, $\sigma _{1}=\bar{\tau}_{h}/\bar{\tau}_{n}\approx1.4817$, $\sigma_{2}=\bar {\tau}_{s}/\bar{\tau}_{n}\approx7.7689$, $\sigma_{3}=\bar{\tau }_{s_{e}}/\bar{\tau}_{n}\approx0.6474$. Note that $\sigma_{1}$, $\sigma_{2}$, and $\sigma_{3}$ are $O(1)$. In (10), all state variables and functions on the right-hand side are $O(1)$ with respect to *ϵ*. The model features two timescales: 1, and $\frac{1}{\epsilon}$. The two voltage variables *v* and $v_{2}$ are fast. All other variables are slow.

Note that the system can be separated into three timescales, with an additional separation between *w* and other gating variables. However, the third timescale does not bring new information to the geometry, as the super-slow manifold is repelling in the relevant parameter range (Appendix 2). Therefore, we use two timescales for our analysis.

With a clear separation of timescales, we utilize geometric singular perturbation theory [29, 30] to study the geometry of the network to understand its dynamics. At the singular limit $\epsilon\to0$, we attain two subsystems: fast and slow.

##### The Fast Subsystem

In the fast subsystem, there are two notable structures, the critical manifold and the fold, that have significant functional implications, which we will show in Sect. 5.2.2.

For convenience, let $x=(v,v_{2})^{T}$, $y=(n,h,s,n_{2},h_{2},s_{e}, w)^{T}$ denote the fast and slow variables, respectively. The dimensionless system (10) can be concisely written as

11 $$ \begin{aligned} \epsilon\dot{x}&=F(x,y), \\ \dot{y} &=G(x,y), \end{aligned} $$

where $F(x,y)=(f_{1}(v,n,h,s,w,s_{e}), f_{2}(v_{2},n_{2},h_{2}))^{T}$, $G(x,y)=(p_{1}(v,n), \frac{1}{\sigma _{1}} p_{2}(v,h), \frac{1}{\sigma_{2}}p_{3}(v,s), p_{4}(v_{2},n_{2}), \frac{1}{\sigma_{1}}p_{5}(v_{2},h_{2}), \frac {1}{\sigma_{3}}p_{6}(v_{2},s_{e}),g(v,w))^{T}$. With a rescaling of the time variable ($\tau^{*}=\tau/\epsilon$) in (11), and then taking the limit $\epsilon\to0$, we obtain the layer problem with respect to the $\epsilon\to0$ limit:

12 $$ \begin{aligned} x'&=F(x,y), \\ y'&=0, \end{aligned} $$

where the prime denotes the derivative with respect to the fast time, $\tau^{*}$. The layer problem describes the fast flow of the singular orbit (the solution at the singular limit). The set of equilibria of (12) is called the critical manifold:

13 $$ C_{0}= \bigl\{ (x,y)\in\mathbb{R}^{9}: F(x,y)=0 \bigr\} . $$

The critical manifold in the $\epsilon\to0$ limit has attracting and repelling subsets. The attracting and repelling subsets are separated by a submanifold, *L*, of fold bifurcations of (12) with respect to *y*, This submanifold *L* is defined by

14 $$ L= \bigl\{ (x,y)\in C_{0}:\det( D_{x}F ) =0 \bigr\} . $$

Because the E-cell equations in (10) evolve independent of the state variables related to the I-cell, the fold condition in (14) simplifies:

15 $$ \det( D_{x}F|_{C_{0}} ) =\det \begin{pmatrix} \frac{\partial f_{1}}{\partial v}| _{C_{0}} & 0 \\ 0 & \frac{\partial f_{2}}{\partial v_{2}}| _{C_{0}} \end{pmatrix} = \frac{\partial f_{1}}{\partial v}\bigg| _{C_{0}} \frac{\partial f_{2}}{\partial v_{2}}\bigg| _{C_{0}}=0. $$

That is, the critical manifold $C_{0}$ has a local turning point whenever either the manifold $\{f_{1}=0\}$ has a fold with respect to *v*, or whenever the manifold $\{f_{2}=0\}$ has a fold with respect to $v_{2}$. We denote the two branches as I-fold ($L_{I}$) and E-fold ($L_{E}$), respectively:

16 $$ \begin{aligned} L_{I}&= \biggl\{ (x,y)\in C_{0}: \frac{\partial f_{1}}{\partial v}\bigg| _{(x,y)}=0 \biggr\} , \\ L_{E}&= \biggl\{ (x,y)\in C_{0}: \frac{\partial f_{2}}{\partial v_{2}}\bigg| _{(x,y)}=0 \biggr\} . \end{aligned} $$

Also notice that the eigenvalues of $D_{x}F|_{C_{0}}$ are $\frac {\partial f_{1}}{\partial v}| _{C_{0}}$ and $\frac{\partial f_{2}}{\partial v_{2}}| _{C_{0}}$, which are both real. Therefore, there is no Hopf bifurcation in the fast subsystem. Depending on the value of slow variables, the (non-fold) equilibrium of the fast system is a sink, a saddle, or a source.

##### The Slow Subsystem

Once the singular orbit is on $C_{0}$, the dynamics is governed by the slow flow, which is described by the reduced problem

17 $$ \begin{aligned} 0&=F(x,y), \\ \dot{y}&=G(x,y). \end{aligned} $$

Note that the reduced problem can be derived by taking the limit $\epsilon\to0$ directly from (11).

We also checked if there exist folded singularities (see Definition 8.1.1 in [29]) on *L*. Folded singularities are generic phenomena in slow/fast systems of the form (10) and give rise to special solutions called canards [29]. Canard solutions stay close to the repelling branch of the slow manifold for some time before moving quickly away. There are models of biological systems where canards are observed [31, 32]. However, in this model, within (and close to) the range of variables and parameters where the singular orbit of the limit cycle typically lies, there are no folded singularities.

Therefore, the fast flow of the limit cycle singular orbit converges to an attracting sheet of $C_{0}$. Then it follows that the slow flow is governed by the reduced problem (17) until it reaches the fold *L*. It is well known that the singular slow flow on $C_{0}$ experiences a finite time blow-up at *L* [29]. Consequently, the limit cycle singular orbit falls off $C_{0}$ and follows the fast dynamics until it reaches an attracting subset of $C_{0}$. The fold corresponds to a firing threshold and it is essential in determining if there is phase-locking, which we will further explain in Sect. 5.2.2. We present an example using the 3D E-cell equations to help the reader who is not familiar with this approach to visualize the critical manifold and the fold in Appendix 3.

#### Folds, Singular Orbits, and Their Implications

In terms of spike order, we can describe sparse entrainment as the I-cell spiking (if it does) after the E-cell spikes. Therefore, in a given cycle, the trajectory always crosses the E-fold before crossing the I-fold, or the trajectory crosses only the E-fold but not the I-fold. We visualize below the I-fold and E-fold of two systems with different $g_{M}$ values to gain insights of how M-current affects the spike order.

Since (10) is nine-dimensional, to visualize the folds, we make projections into $(v, v_{2}, s)$ space via approximating the membrane current gating variables as functions of *v* and $v_{2}$. We take the following projections based on the separation of timescales:

18 $$ \begin{aligned} n&=n_{\infty}(v), \quad\quad h=h_{\infty}(v),\quad\quad n_{2}=n_{\infty}(v_{2}), \\ h_{2}&=h_{\infty}(v_{2}),\quad\quad s_{e}=q(v_{2}), \end{aligned} $$

where $q(v_{2})$ satisfies $p_{6}(v_{2}, q(v_{2}))=0$ in (10).

We chose to project these variables because they are the next five fastest variables besides *v* and $v_{2}$. These projections will make $p_{1}(v,n)=p_{2}(v,h)=p_{4}(v_{2},n_{2})=p_{5}(v_{2},h_{2})=p_{6}(v_{2},s_{e})=0$ in (10). After applying (18), the projected fold is now in $(v,v_{2},s,w)$ space. We also fixed *w* (at some typical value before a spike; see Appendix 5 for details) to visualize a slice of this four-dimensional space in 3D $(v,v_{2},s)$-space, because *w* is the slowest variable.

**Fig. 7:**
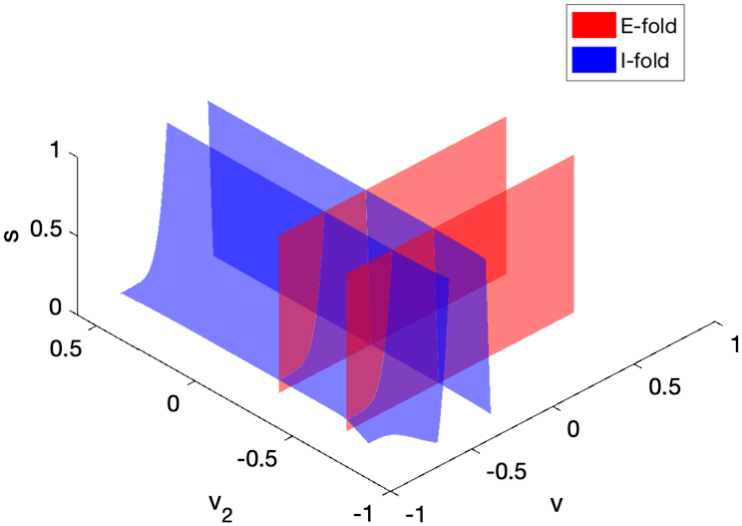
Approximated position of the fold *L* of the network model (10). Both branches $L_{I}$ and $L_{E}$ have two sheets. The sheets at lower *v*, $v_{2}$ values represent the firing thresholds of the I-cell and E-cell, respectively

We plotted the I-fold and the E-fold in the projected space in Fig. 7. Notice that each fold has two sheets, one (at lower voltage value) represents the firing threshold and the other is where the voltage returns after a spike. The two-sheet fold structure suggests that the I-cell subsystem, although having 5 dimensions, has a similar geometric structure for spiking as the 3D E-cell subsystem, where there are two lines of fold points on the critical manifold, one of which is the firing threshold (shown in Appendix 3).

**Fig. 8:**
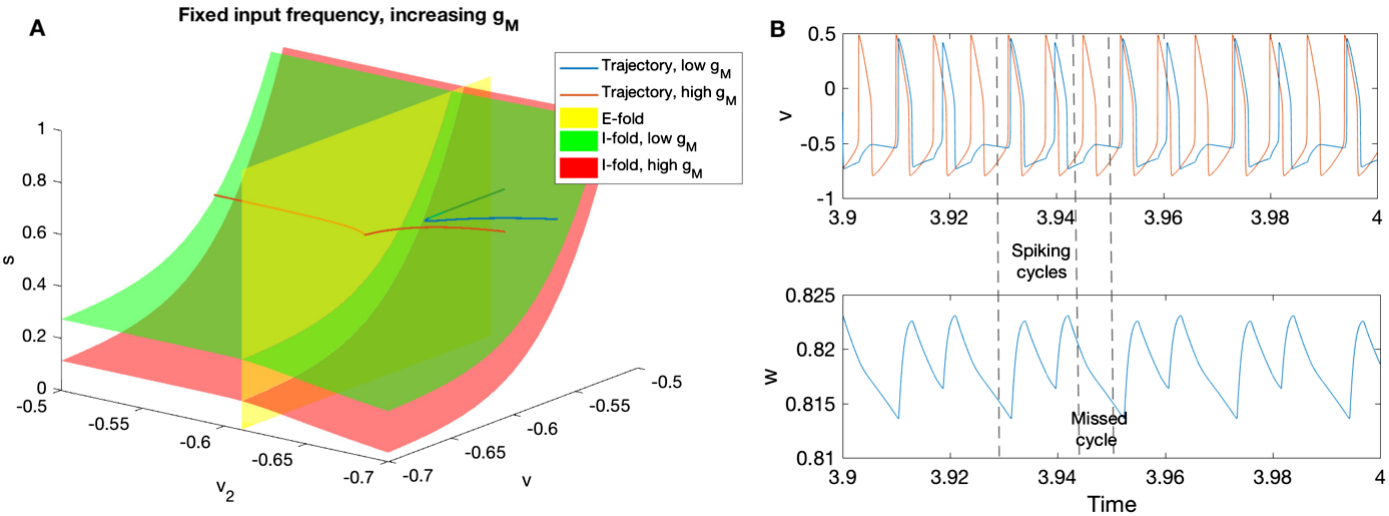
**A**: Increasing $g_{M}$ moves the I-fold in the positive *v* direction, equivalent to raising the I-cell threshold. The singular trajectories are approximated by setting $\epsilon=0.001$ in (10). Folds are approximated by equations (18), and fixing $w=0.74$ for the green (low $g_{M}$) I-fold, and $w=0.7$ for the red (high $g_{M}$) I-fold. Other parameters used to generate this figure can be found in Appendix 4. **B**: The voltage traces of the I-cell (blue) and E-cell (red) are shown in the top panel. Parameter *J* is increased to 20 to show sparse entrainment. Other parameters are the same as the high $g_{M}$ case in **A**. The M-current gating variable *w* is shown in the bottom panel. It builds up during spiking cycles and decays during missed cycles

In Fig. 8A, we plotted the folds of two systems with different $g_{M}$ values. Their I-cell subsystems have the same natural frequency (at $\epsilon=0.001$); the two E-cell subsystems are identical. There is sparse entrainment in the high $g_{M}$ system but not in the low $g_{M}$ system. Appendix 4 explains how and why we chose these parameter values. We find that increasing $g_{M}$ moves the I-fold towards the positive *v* direction. Functionally, the firing threshold of the I-cell with high M-current is higher than the one with low M-current, although these two cells have the same excitability (natural frequency).

As the trajectories approach the folds, the low $g_{M}$ (blue) trajectory hits the (green) I-fold before reaching the E-fold: the I-cell spikes before the input arrives. By contrast, with high $g_{M}$, the corresponding (red) I-fold is farther away: the trajectory encounters the E-fold first. That is, the I-cell with high $g_{M}$ fires after the input. Moreover, the velocity of high $g_{M}$ trajectory (red) is slightly slower in the *v* direction than the velocity of low $g_{M}$ trajectory (blue), which can be inferred directly from (10). The changes in the I-fold position and trajectory traveling speed result in the change of the first fold encountered by the trajectory.

As a result, with low M-current, the I-fold is closer to the trajectory than in the high M-current case. Therefore, the trajectory may cross the I-fold first, causing the I-cell firing before the external pulse arrives. In contrast, the trajectory with high M-current always crosses the E-fold first during each input cycle. After that, it may or may not cross the I-fold. Equivalently, in the simulation, we observe sparse entrainment (Sect. 3, last row of Fig. 3A), where the I-cell spikes are always in alignment with the input with some missed cycles. During cycles that the I-cell does not spike, the M-current gating variable *w* decreases. The decrease in *w* causes the I-fold to move towards to the trajectory. However, the amount *w* decreases during the missed cycle is not more than the amount it increased during the spiking cycles (Fig. 8B). Therefore, when there is sparse entrainment, the I-fold moves further away from the trajectory during consecutive spikes, but shifts back during missed cycles. By the next time the I-cell is ready to spike again, the I-fold is still far enough from the trajectory such that the trajectory crosses the E-fold first. The sparse entrainment to gamma input in the presence of an M-current is very robust and is observed even for high gamma input frequencies (up to 80 Hz, the maximum input frequency simulated using System (1)).

### Bistability of the I-Cell

We have seen in Sect. 5.1 that M-current helps the oscillator phase-lock to a rhythm slower than its natural frequency and in Sect. 5.2 that M-current allows the oscillator to respond to input faster than the $1:1$ phase-locking range through sparse entrainment. Here, we present a special case where the I-cell with M-current is able to $1:1$ phase-lock to an arbitrarily slow rhythm, due to bistability.

#### M-Current Allows the I-Cell with Low Excitation to Phase-Lock to an Arbitrarily Slow Input Through Bistability

**Fig. 9:**
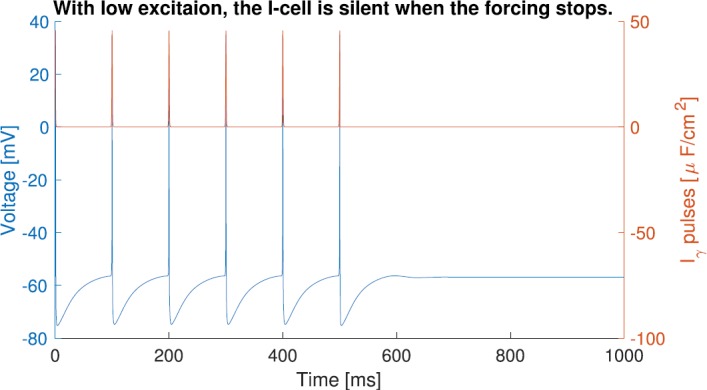
The I-cell with M-current of natural frequency 16 Hz can phase-lock to a 10 Hz input. It becomes silent when the forcing stops. The voltage trace of the I-cell is in blue. External forcing is in red

With low background excitation ($I_{\mathit {ton}}<5.5$ *μ*A/cm^2^), the I-cell with M-current can phase-lock to an arbitrarily slow input. Figure 9 shows a cell with M-current with 16 Hz natural frequency ($I_{\mathit {ton}}=5$) being $1:1$ phase-locked to a 10 Hz input for the first 500 ms. After 500 ms, the forcing is turned off. The cell remains silent instead of returning to its natural 16 Hz oscillation. The internal oscillation of this cell can be turned off by external pulses. Being in the silent state enables the cell to $1:1$ phase-lock to arbitrarily slow external pulses.

**Fig. 10:**
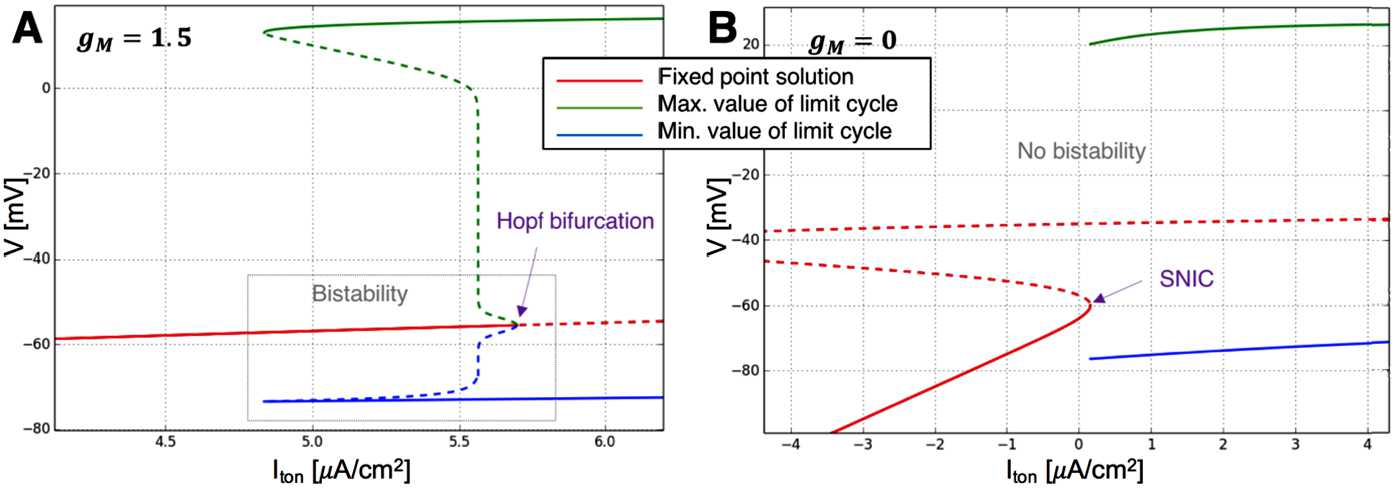
Bifurcation structure of the I-cell (without gamma or theta input, i.e. System (1) subject to (3) with $a=b=0$) with respect to $I_{\mathit {ton}}$ when **A**: $g_{M}=1.5$ mS/cm^2^ and **B**: $g_{M}=0$ mS/cm^2^. When $g_{M}=1.5$ mS/cm^2^, the stable fixed point ends at a subcritical Hopf bifurcation. A family of stable limit cycles is born at a saddle-node bifurcation of cycles. There is a bistability window of $I_{\mathit {ton}}$ values such that a stable fixed point and a stable limit cycle coexist. When $g_{M}=0$ mS/cm^2^, the stable fixed points terminate and a family of stable limit cycles is born at a SNIC bifurcation

Using the numerical continuation software package AUTO [33], we constructed the bifurcation diagram (Fig. 10A) of the I-cell with M-current (1) but without gamma or theta forcing, with tonic current as the bifurcation parameter. With the M-current and a low excitation level, a stable limit cycle coexists with a stable fixed point in the bifurcation diagram of the I-cell. A family of unstable limit cycles is born from a subcritical Hopf bifurcation (at $I_{\mathit {ton}}\approx5.6$ *μ*A/cm^2^). The unstable limit cycle and the stable limit cycle meets at a saddle node of periodics. The I-cell ((1) subject to (3) with $a=b=0$) with M-current ($g_{M}=1.5$ mS/cm^2^) has an interval of bistability between the $I_{\mathit {ton}}$ values where the saddle node of periodics and the subcritical Hopf bifurcations occur. This is the range of excitation level with which we can observe the shutting off of internal oscillations of the I-cell. The bifurcation diagram indicates that the I-cell with M-current is a Type II neuron, i.e., the stable limit cycle starts at a nonzero frequency [28, 34].

Without the M-current ($g_{M}=0$ mS/cm^2^, Fig. 10B), the I-cell does not manifest bistability. The cell’s stable fixed point terminates at a saddle node on invariant circle (SNIC) bifurcation and a stable limit cycle is born. This family of limit cycles starts at infinite period, i.e., zero frequency. Hence, the cell without M-current is a Type I neuron [28, 34]. The left bifurcation diagram transitions smoothly into the right as $g_{M}$ decreases. The Hopf bifurcation point becomes a SNIC through a Bogdanov–Takens bifurcation.

Therefore, with baseline parameters, bistability exists when there is an M-current and the excitation is within the range where a fixed point and a stable limit cycle coexist. These are the conditions of the I-cell being entrained by arbitrarily slow inputs.

#### Possible Ways to Expand the Bistability Range

In Fig. 10A, the bistability region under default parameters spans over about 0.86 *μ*A/cm^2^, which is quite small. We explore how changes in the parameters can affect the size of this bistability window. In AUTO, we continue the Hopf bifurcation point and the starting point of the stable limit cycle in parameters $g_{L}$, $g_{K}$, $g_{\mathit {Na}}$, $g_{s}$, and $g_{M}$.

**Fig. 11:**
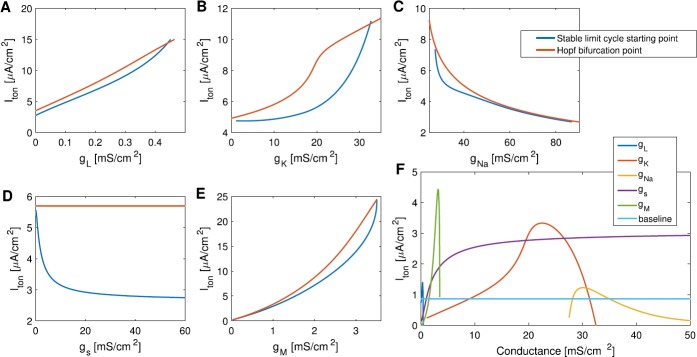
**A**–**E**: The change in the starting point of the stable limit cycle and the Hopf bifurcation point as parameters $g_{L}$, $g_{K}$, $g_{\mathit {Na}}$, $g_{s}$, and $g_{M}$ change. The vertical distance between the blue and red curves is the size of the bistability window. **F**: The size of the bistability window as parameters $g_{L}$, $g_{K}$, $g_{\mathit {Na}}$, $g_{s}$, and $g_{M}$ change. The baseline at around 0.86 *μ*A/cm^2^ is the size of the bistability window with default parameter values. Changes in $g_{K}$, $g_{s}$, and $g_{M}$ increase the bistability window size significantly. Increasing the maximal M-current conductance $g_{M}$ to approximately 3.2066 mS/cm^2^ has the most dramatic effect

We show below that a reasonable increase in the maximal conductance of the M-current or the potassium current will expand the bistability window, while other parameters either do not expand the window much, or need to be increased dramatically to achieve the effect. Figures 11A–E show how the Hopf bifurcation point and the stable limit cycle starting point move as we move away from the baseline values for the maximal conductances ($g_{L}$, $g_{K}$, $g_{\mathit {Na}}$, $g_{s}$, and $g_{M}$). The two points move at different rates. It is not clear when the size of the bistability window is at its maximum from Figs. 11A–E. Therefore, we compute the vertical distance between the red curve and the blue curve for each subplot and put them together in one graph (Fig. 11F). Changes in $g_{K}$, $g_{s}$, and $g_{M}$ increase the bistability window size significantly. However, $g_{s}$ needs to be at least 10 times of its baseline value to make the bistability window size larger than 2.5 *μ*A/cm^2^, which is the value near the plateau of the $g_{s}$ curve in Fig. 11F. When $g_{M}\approx 3.2066$ mS/cm^2^, a little more than doubling the baseline value, the bistability window size is at its maximum (around 4.4303 *μ*A/cm^2^). The size of the bistability window is also larger than 3 *μ*A/cm^2^ when 20.1 mS/cm^2^$< g_{K}<25.8$ mS/cm^2^, less than tripling the baseline value ($g_{K}=9$ mS/cm^2^). Therefore, increasing the maximal conductance of the M-current or the potassium current can most easily make the I-cell be bistable subject to a larger range of background excitation.

Bistability is of functional significance as it permits an otherwise oscillating neuron to be entrained by an arbitrarily slow spike train. The phase-locking range is dramatically expanded. The phase-locking is also more robust: if the forcing frequency changes abruptly (but within the $1:1$ phase-locking range), the cell can follow without delay.

## h-Current Also Allows the Cell to Phase-Lock to Rhythms Slower than Its Natural Frequency, but Spikes Occur Before Inputs

In models of hippocampal gamma and theta oscillations, the intrinsic theta timescale comes from an intrinsic membrane current, often an h-current or an M-current [3, 4, 18, 35, 36]. The M-current and the h-current are functionally similar in that they both oppose changes in voltage in response to an applied current at a slow timescale. The h-current is an inward current that activates with hyperpolarization, whereas the M-current is an outward current that decreases with hyperpolarization.

**Fig. 12:**
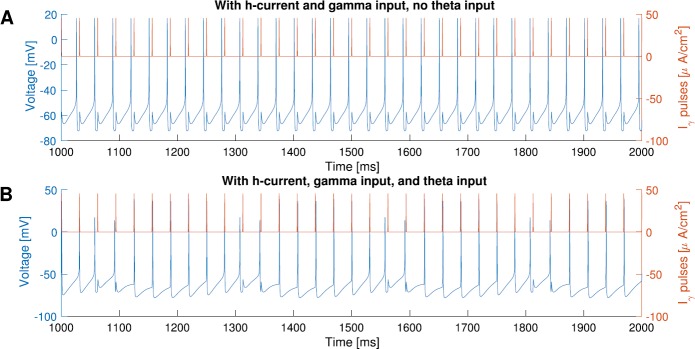
Simulations of (1) with h-current instead of M-current. The expression of the h-current used here is the same as the one used in [37]. **A**: The cell with h-current ($g_{h}=2$, $I_{\mathit {ton}}=-6$, $b=0$) can $1:1$ phase-lock to 32 Hz input while the cell’s natural frequency is 34.5 Hz. But the spikes (blue) precede the input (red). **B**: Same as **A**, but add the theta input and reduce the tonic current so the natural frequency is the same at 34.5 Hz ($g_{h}=2$, $I_{\mathit {ton}}=-10$, $b=4$) at the peak of theta input. The cell with h-current (blue) has the same firing frequency as the input (red). Unlike the cell with M-current, where the spikes always occur after the input (Fig. 1A), some spikes of the cell with h-current occur before the input

We found that replacing the M-current by the h-current (Appendix 6) can reproduce some but not all results. Like the M-current, the h-current allows the cell to $1:1$ phase-lock to inputs slower than the cell’s natural frequency. Figure 12A shows a cell with h-current with 34.5 Hz natural frequency can $1:1$ phase-lock to 32 Hz input. In fact, it is able to $1:1$ phase-lock to inputs as slow as 30 Hz. However, all spikes in Fig. 12A occur consistently before the input, whereas with M-current, the spikes always occur after the input. As a result, when the theta input (sinusoidal current) is added, spikes that occur near the peak of theta input precede the external pulses, yet the spiking frequency of the cell with h-current is the same as the external pulses (Fig. 12B). We were not able to obtain spikes occurring after inputs using the h-current for a wide range of strengths of the h-current simulated. It is unclear what mathematical aspect is responsible for the differences we observe in the systems with M-current and with h-current. It could be because the disparity between the M-current being activated after a spike and the h-current being deactivated after a spike. More work needs to be done to understand the difference and its functional implications.

## Discussion

In this paper, we consider a small model network consisting of a single cell with an inhibitory synapse. The network has both gamma (inhibitory synapse) and theta (M-current) timescales, and is forced by a combination of spiking gamma input and sinusoidal theta input. The M-current allows the network to $1:1$ phase-lock to a wider range of frequencies than a network without the M-current but with matched natural frequency. It also provides homeostasis against the oscillating theta input, making the frequency of the network less variable over a theta cycle. However, this homeostatic effect works only when the sinusoidal forcing is of theta or slower frequencies. Additionally, when responding to pulsatile gamma inputs too fast to follow cycle by cycle, the system with the M-current exhibits sparse entrainment. By contrast, the system without the M-current (but with the same natural frequency) reacts to faster gamma inputs by phase walk-through.

The reduced model with artificial spikes reveals that the external pulses push the reset point of the limit cycle to a higher place on the slow manifold than when there is no external pulse, effectively extending the interspike interval. To understand sparse entrainment, which requires studying both inputs and outputs, we utilized geometric singular perturbation theory on the E-I network model. We showed that the timing of the singular orbit crossing the branches of the fold is essential to determining whether there is entrainment in the system at its singular limit. The position of the fold is influenced by the maximal conductance of the M-current and therefore alters the spike order. The M-current also changes the bifurcation structure such that the system becomes bistable, which further increases the robustness of the system’s ability to be entrained to external pulses. Bifurcation analysis and numerical continuation show that increasing the maximal conductance of the M-current or the potassium current expands the bistability region significantly.

In the present work, we explored how internal network theta and gamma timescales interact with external theta and gamma forcing. Interestingly, we found that it is the intrinsic network theta timescale that allows the intrinsic gamma frequency spiking to coordinate precisely with the excitatory gamma forcing input. Note that although the internal theta timescale is also the subthreshold resonance frequency of the cell (due to the M-current), this does not automatically imply suprathreshold resonance or precise response spiking [38]. The phenomenon we are studying in this paper is the phase-locking of spikes, not subthreshold resonance. Precisely timed spiking is critical to spike-time-dependent plasticity, a mechanism thought to be important to learning and memory [14].

To the best of our knowledge, this is the first demonstration of the phenomenon that the M-current, which has a theta timescale, contributes to the precise coordination of spikes of gamma frequency. Since the M-current is a non-inactivating potassium current with a slow decay time constant, it is active in the subthreshold range of membrane voltages and is able to influence the membrane potential of neurons during the interspike interval [28, 39]. Our analysis of how the M-current allows the cell to be entrained by rhythms slower than the cell’s natural frequency indicates that the M-current works to prolong the duration of the intrinsic network interspike interval when excitatory gamma forcing is present. Prolongation of the intrinsic network interspike interval allows excitatory gamma input to force the network to entrain to a lower gamma frequency than its intrinsic gamma oscillation frequency.

The prolongation of the intrinsic network interspike interval by gamma forcing is observed from two perspectives. One is the higher reset point after each spike triggered by external pulses indicated by the reduced model. The other perspective is from the timing of spike onset inferred from the geometry of the network system. We saw a relative shift in the positions of the I-cell fold structure with respect to the singular orbit of the limit cycle. The M-current shifts the I-cell fold further away than the E-fold from the singular orbit, thus allowing the forcing input (E-cell input) to pace the system. Here we do not consider folded singularities on the fold structures because, although folded singularities can constrain dynamics near them; in our case there were no such singularities near the relevant physiological parameters.

We also observed that the homeostatic effect of M-current is gradually reduced as the frequency of the sinusoidal current increases in Sect. 4. The fastest sinusoidal current that can be imposed onto the cell while preserving the cell’s entrainment to gamma pulses seems to be related to the subthreshold resonance frequency of the cell as a result of having the M-current. However, we currently do not have a way to mathematically explain the connection between the two, since it is not clear how the subthreshold resonant properties of individual cells are communicated to the spiking regime in the presence of a limit cycle. Further research is needed to address this question, not only for the specific case studied here, but also in a general context.

Additionally, we find that the M-current provides a region of bistability to the network system such that the network can phase-lock to an arbitrarily slow forcing input by turning off its internal oscillation. Previous works have shown that the M-current alters the neuron’s firing mechanism. In [40], Ermentrout et al. reported that, without the M-current, the neuron’s stable limit cycle is created at a saddle-node point. With the M-current, however, the stable fixed point loses its stability at a Hopf bifurcation. Thus, the addition of the M-current changes the neuron from Type I to Type II. Acker et al. [37] also reported on the switch of neuron type due to the addition of a slow resonating current (an h-current or a slow, non-inactivating potassium current, e.g., M-current). Moreover, in Sect. 4.4.2 of [41], Ermentrout and Terman also addressed how adding the M-current allows a SNIC to transition to Hopf through a Bogdanov–Takens bifurcation. However, these results do not discuss whether there is bistability, or the implication of bistability on the system (allowing the cell to be entrained by an arbitrarily slow rhythm), as we have shown here.

Last, we addressed what happens when we replace the M-current by the h-current. While both M-current and h-current are able to allow the cell to be entrained by a rhythm slower than its natural frequency, the resulting spike orders are different. With an M-current, the I-cell follows the input, while the I-cell precedes the input with an h-current. It suggests that the M-current and the h-current have very different functional implications for plasticity. In the case of the M-current, the cell spiking after the input would strengthen the synaptic connection; whereas with an h-current, the synaptic connection would be depressed due to the cell spiking before the input [14]. We were able to reproduce Fig. 12 using a reduced model with h-current (similar to the one used in Sect. 5.1), but the phase plane of the reduced model with h-current does not offer obvious explanations of the spike order difference observed in the models with M-current and with h-current.

The major finding of this work is that the theta timescale properties of the M-current allow this E-I network to precisely coordinate with external forcing on a gamma timescale. This work shows how the interaction of currents on different timescales can be of functional importance in network level phenomena.

## Figures and Tables

**Fig. 13:**
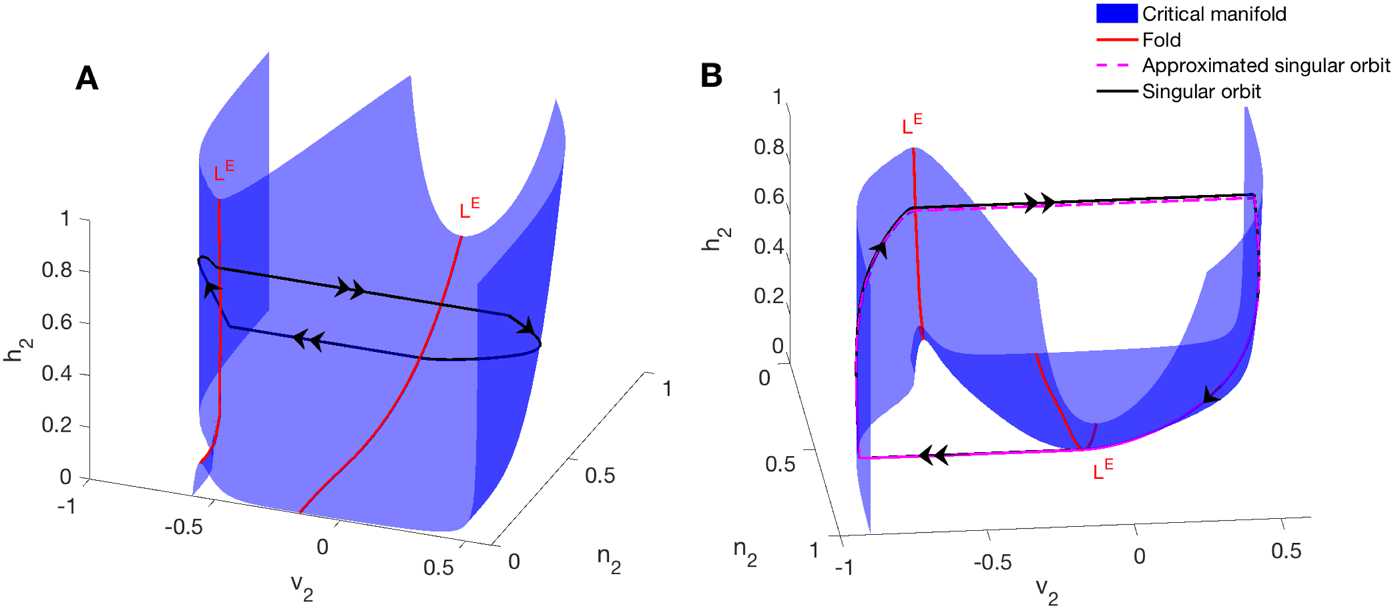
Two perspectives of the E-cell critical manifold $C_{0}^{E}$, fold $L^{E}$, and the singular orbit of the limit cycle. **A**: Critical manifold $C_{0}^{E}$ has a cubic shape. $L^{E}$ separates $C_{0}^{E}$ into two outer attracting sheets and one inner repelling sheet. **B**: Another perspective of $C_{0}^{E}$. It gives a better idea about the position of the singular orbit relative to $C_{0}^{E}$ and $L^{E}$. The slow part of the orbit lies on $C_{0}^{E}$. The switch from the slow flow to the fast flow happens exactly at the fold $L^{E}$, which represents the physiological firing threshold. We also used $\epsilon =0.001$ in (20) to approximate the singular orbit numerically (dashed magenta line). It stays very close to the singular orbit

**Fig. 14:**
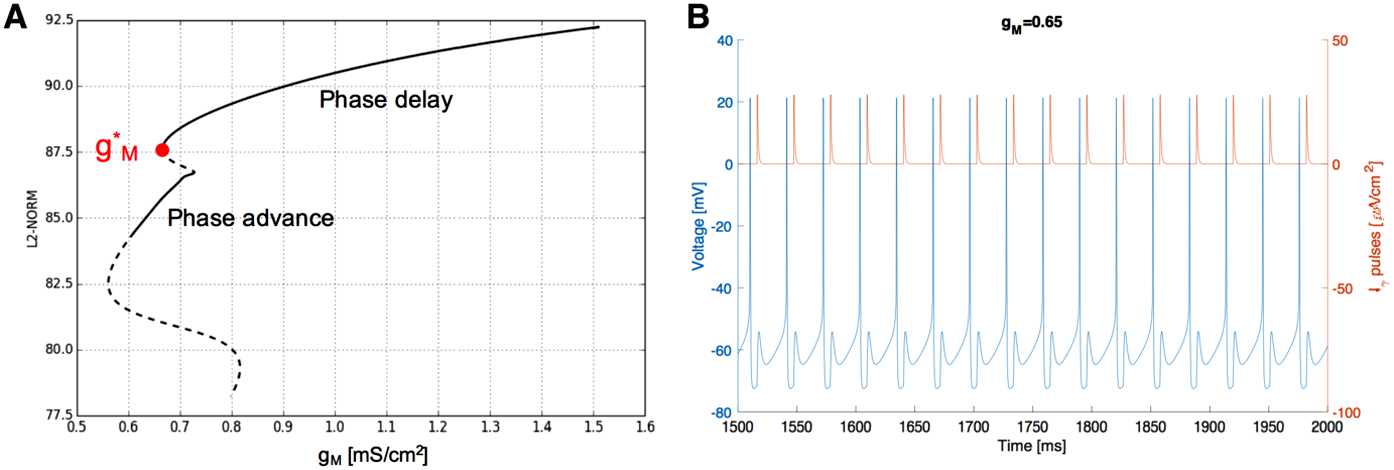
**A**: Bifurcation diagram of the stable limit cycle of (10) with respect to parameter $g_{M}$. Stable limit cycles are represented by solid curves, while unstable limit cycles are drawn as dashed curves. The stable limit cycle at $g_{M}=1.5$ mS/cm^2^ continues as $g_{M}$ decreases and terminates at $g_{M}^{*} \approx0.67$ mS/cm^2^ (phase delay limit cycles, top branch of stable limit cycles). The bottom branch of stable limit cycles corresponds to $1:1$ phase-locking when the I-cell fires consistently before the E-cell (phase advance limit cycles). **B**: Simulation of (10) with units when $g_{M}=0.65$ mS/cm^2^. The stable limit cycle is on the bottom branch of the stable limit cycles in the bifurcation diagram in **A**. The I-cell does $1:1$ phase-lock to the E-cell. However, I-cell consistently spikes before the E-cell does, not immediately after the E-cell’s spikes. The voltage trace of the I-cell is in blue. The external pulses are in red

**Fig. 15:**
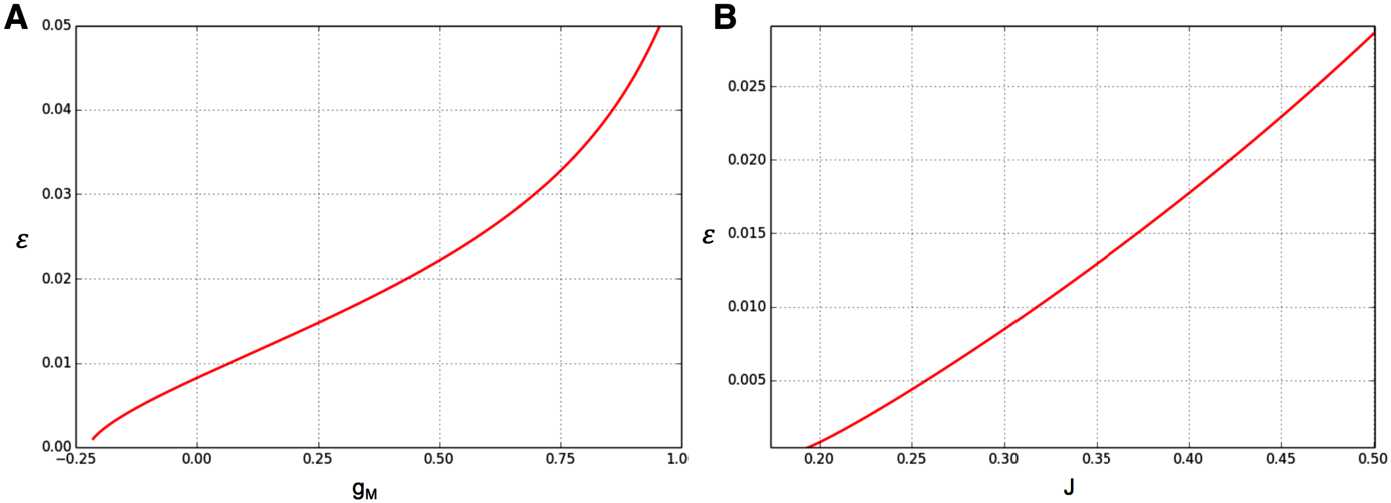
Two-parameter continuation of the boundary point of $1:1$ phase-locking range of (10) in AUTO. **A**: As $\epsilon\to0$, $g_{M}^{*}$ moves to a negative value. **B**: As $\epsilon\to0$, $1:1$ phase-locking range begins when $J\approx0.2$

**Fig. 16:**
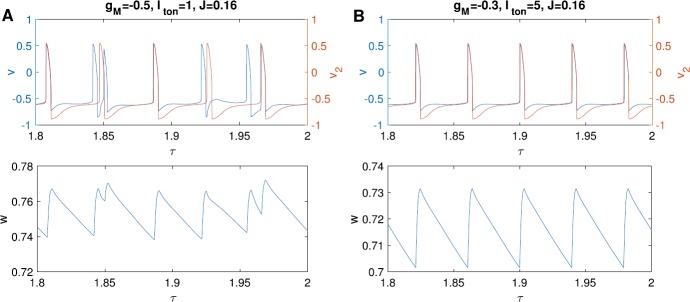
Simulations results of (10) with $\epsilon=0.001$ to approximate the singular orbit. **A**: Using parameters $g_{M}=-0.5$, $I_{\mathit {ton}}=1$, $J=0.16$, $w\approx0.74$ before the I-cell spikes. **B**: Using parameters $g_{M}=-0.3$, $I_{\mathit {ton}}=5$, $J=0.16$, $w\approx0.7$ before the I-cell spikes. Top row: voltage traces of the I-cell (blue) and the E-cell (red). Bottom row: the trace of M-current gating variable *w*

## Appendix 1: Parameters, Units, and Functions in the Mathematical Model

Here we provide the parameter values and expressions of auxiliary functions used in the mathematical model. In particular, Table A.1 contains baseline parameter values for (1) and (7). Expressions for infinity and *τ* functions in Eq. (1) can be found in Table A.2. Functions and parameters that describes the forcing terms are given in Table A.3.

## Appendix 2: Three-Timescale Separations

We showed in Sect. 5.2.1 that (1) has two timescales through non-dimensionalization. However, the system can be separated further into three timescales as follows.

19 $$\begin{aligned} \epsilon_{2}\delta\frac{dv}{d\tau}&=\bar {g}_{L}(e_{L}-v)+\bar{g}_{K}n^{4}(e_{K}-v)+ \bar{g}_{\mathit {Na}}m^{3}_{\infty}(k_{v} v)h(e_{\mathit {Na}}-v)+\bar{g}_{s} s(e_{s}-v) \\ & \quad {} +\bar{g}_{M}w(e_{M}-v)+\bar{g}_{f}s_{e}(v_{e}-v)+ \frac{I_{\mathit {ton}}}{k_{v} g_{\mathit {ref}}}, \\ \delta\frac{dn}{d\tau}&=\frac{n_{\infty}(k_{v} v)-n}{\tau_{n}(k_{v} v)/\bar{\tau}_{n}}, \\ \sigma_{1}\delta\frac{dh}{d\tau}&=\frac{h_{\infty}(k_{v} v)-h}{\tau _{h}(k_{v} v)/\bar{\tau}_{h}}, \\ \sigma_{2}\delta\frac{ds}{d\tau}&=\frac{1}{2} \biggl[ 1+ \tanh \biggl( \frac{k_{v} v}{4} \biggr) \biggr] \frac{1-s}{\tau _{r}/\bar{\tau}_{s}}- \frac{s}{\tau_{d}/\bar{\tau}_{s}}, \\ \frac{dw}{d\tau}&=\frac{w_{\infty}(k_{v} v)-w}{\tau_{M}(k_{v} v)/k_{t}}, \\ \epsilon_{2}\delta\frac{dv_{2}}{d\tau}&=\bar{g}_{L}(e_{L}-v_{2})+ \bar{g}_{K}n_{2}^{4}(e_{K}-v_{2})+ \bar{g}_{\mathit {Na}}m^{3}_{\infty}(k_{v} v_{2})h_{2}(e_{\mathit {Na}}-v_{2}) \\ & \quad {} +\frac{J}{k_{v} g_{\mathit {ref}}}, \\ \delta\frac{dn_{2}}{d\tau}&=\frac{n_{\infty}(k_{v} v_{2})-n_{2}}{\tau_{n}(k_{v} v_{2})/\bar{\tau}_{n}}, \\ \sigma_{1}\delta\frac{dh_{2}}{d\tau}&=\frac{h_{\infty}(k_{v} v_{2})-h_{2}}{\tau_{h}(k_{v} v_{2})/\bar{\tau}_{h}}, \\ \sigma_{3}\delta\frac{ds_{e}}{d\tau}&=\frac{1}{2} \biggl[ 1+ \tanh \biggl( \frac{k_{v} v_{2}}{4} \biggr) \biggr] \frac{1-s_{e}}{\tau _{r_{e}}/\bar{\tau}_{s_{e}}}- \frac{s_{e}}{\tau_{d_{e}}/\bar{\tau}_{s_{e}}}, \end{aligned}$$

where $\epsilon_{2}=\frac{C}{g_{\mathit {ref}} \bar{\tau}_{n}}\approx0.0863 \ll1$, $\delta=\bar{\tau}_{n}/k_{t}\approx0.0106 \ll1$, and all other variables are defined as in Sect. 5.2.1. Here, all state variables and functions on the right-hand side are $O(1)$ with respect to $\epsilon_{2}$ and *δ*. System (19) features a cascade of timescales: 1, $\frac{1}{\delta }$, and $\frac{1}{\epsilon_{2}\delta}$. The two voltage variables *v* and $v_{2}$ are fast. All gating variables except *w* are slow. The M-current gating variable *w* is super-slow.

With three timescales, there two ways to take the singular limit: $\epsilon_{2}\to0$ and $\delta\to0$. The fast subsystem is the same as in Sect. 5.2.1 by taking $\epsilon_{2}\to0$. As the singular orbit lands on $C_{0}$, the critical manifold, it switches to the slow flow, which itself is a slow/fast system with timescales separated by *δ*. Define the super-slow manifold as

$$ C_{0}^{ss}= \bigl\{ (x,y)\in\mathbb{R}^{9}: F(x,y)=G_{1}(x,y)=0 \bigr\} , $$

where $G_{1}$ is a vector of the first six elements of *G* (see Sect. 5.2.1). Note that $C_{0}^{ss}$ is one-dimensional and repelling in the parameter range we study. This timescale separation by *δ* does not bring new geometric insight about the slow flow on $C_{0}$. Thus, we focus only on the singular limit $\epsilon_{2}\to0$, which is the same as the singular limit $\epsilon\to0$ in (10).

## Appendix 3: Example: E-Cell

Here we use the E-cell subsystem to provide an example of visualizing the geometry of the system at its singular limit. The low dimensionality of the E-cell system allows us to easily visualize how the fold acts as a firing threshold. The dimensionless equations of the E-cell are

20 $$ \begin{aligned} \epsilon\delta\frac{dv_{2}}{d\tau}&=f_{2}(v_{2},n_{2},h_{2}), \\ \delta\frac{dn_{2}}{d\tau}&=p_{4}(v_{2},n_{2}), \\ \sigma_{1}\delta\frac{dh_{2}}{d\tau}&=p_{5}(v_{2},h_{2}). \end{aligned} $$

In the $\epsilon\to0$ limit, the critical manifold of the E-cell is

21 $$ C_{0}^{E}= \bigl\{ (v_{2},n_{2},h_{2}):f_{2}(v_{2},n_{2},h_{2})=0 \bigr\} . $$

The fold on $C_{0}^{E}$ with respect to the slow variables $n_{2}$, $h_{2}$ is

22 $$ L^{E}= \biggl\{ (v_{2},n_{2},h_{2})\in C_{0}^{E}: \frac{\partial f_{2}}{\partial v_{2}}=0 \biggr\} . $$

We use superscript $L^{E}$ to distinguish the fold of the E-cell subsystem (20) from the E-fold branch $L_{E}$ of the network model (10).

In Fig. 13, we show the E-cell critical manifold $C_{0}^{E}$ (blue surface), E-cell fold $L^{E}$ (red lines), and the singular orbit (black curve) from two perspectives. The slow part of the singular orbit moves on $C_{0}^{E}$ and the orbit jumps at $L^{E}$ to the opposite attracting sheet of $C_{0}^{E}$. Therefore, one branch of the fold $L^{E}$ (the one that the singular orbit crosses as $v_{2}$ increases) is equivalent to a physiological firing threshold. This allows us to identify a geometric structure, $L^{E}$, which can be used as the separation between spiking and non-spiking behavior of the neuron.

The true singular orbit of the limit cycle can be well approximated by a numerically simulated one. In Fig. 13B, the numerically approximated singular orbit is obtained by simulating (20) with $\epsilon=0.001$. Note that this approximated orbit stays very close to the real singular orbit of the limit cycle, which can be constructed by concatenating the slow flow and the fast flow. We used the approximated singular orbit instead of the real singular orbit in Fig. 8. We do this because for fair comparison, we need to adjust $I_{\mathit {ton}}$ to ensure the I-cells with and without M-current have the same natural frequency. However, at the limit $\epsilon\to0$, frequency loses its physical meaning. The $\epsilon=0.001$ is used only for approximating singular orbits. All other geometric structures (i.e. critical manifold, folds) are defined at the $\epsilon\to0$ limit.

## Appendix 4: Choice of $g_{M}$ and *J* in Fig. 8

Figure 8 provides a visualization of the multidimensional geometric structure of the fold manifolds of (10), which is essential in understanding how the M-current affects the phase-locking. Since the fold manifolds are six-dimensional, it requires careful choices of approximations and projections to present them in a space of lower dimension (the three-dimensional $(v,v_{2},s)$ space). In this section, we list the values of $g_{M}$ and *J* used to generate Fig. 8 and explain why we choose them, for the purpose of reproducing the results.

Here we define a particular value of $g_{M}$ as the boundary between phase-locking and not phase-locking. We have seen in Fig. 1 that the I-cell with M-current ($g_{M}=1.5$ mS/cm^2^) can phase-lock to gamma pulses, while the I-cell without M-current ($g_{M}=0$ mS/cm^2^) cannot. There exists a value $g_{M}^{*}$ such that the I-cell can phase-lock to the external pulses if and only if $g_{M}> g_{M}^{*}$. In the E-I model (10), we find, numerically, that $g_{M}^{*}\approx0.67$ mS/cm^2^. This was done by first finding the stable limit cycle of (10) with original parameters. Next, in AUTO, we continue this stable limit cycle in parameter $g_{M}$ to obtain the bifurcation diagram in Fig. 14. The stable limit cycle at $g_{M}=1.5$ mS/cm^2^ continues as $g_{M}$ decreases and terminates at $g_{M}^{*}\approx 0.67$ mS/cm^2^.

Notice that there is another branch of stable limit cycles that is not connected with the curve of stable limit cycles continued from $g_{M}=1.5$ mS/cm^2^. Although this set of stable limit cycles also represents $1:1$ phase-locking between the I-cell and the E-cell, the I-cell consistently spikes before the E-cell instead of right after the E-cell (Fig. 14B). We call this the “phase advance $1:1$ phase-locking.” This regime occurs in a much smaller parameter window compared with the other regime, which we saw in Fig. 1A (call it the “phase delay $1:1$ phase-locking”). Moreover, both phase advance and phase delay $1:1$ phase-locking stable limit cycles exist when 0.67 mS/cm^2^$< g_{M}<0.72$ mS/cm^2^. It depends on the initial condition to which regime the system will converge. Since the phase delay $1:1$ phase-locking regime is more typical, we consider the value where the phase delay $1:1$ phase-locking breaks as the boundary between “I-cell follows E-cell” and “I-cell cannot follow E-cell.” Since we consider the $\epsilon\to0$ limit in Sect. 5.2.1, we continue the boundary $g_{M}^{*}$ in two parameters $g_{M}$ and *ϵ* in AUTO. As $\epsilon\to0$, the boundary $g_{M}^{*}$ shifts to negative values (Fig. 15A). Although it is out of the physiologically reasonable range, it does not affect the mathematical analysis.

The network model (10) and its singular limit do not have the same boundary value $g_{M}^{*}$. The parameter *J* of the E-cell also changes in the singular limit. We know from Fig. 14A that the $1:1$ phase-locking range of (10) starts at $g_{M}^{*}\approx 0.67$ mS/cm^2^ and $J=0.5$ *μ*A/cm^2^. Figure 15A shows that $g_{M}^{*}$ shifts to negative values when $\epsilon\to0$. For a given input (fixed *J*), there exists a boundary value $g_{M}^{*}$ where $1:1$ phase-locking starts. Similarly, given a fixed $g_{M}$, there exists a boundary value for *J* (controlling the input frequency) where $1:1$ phase-locking starts. As approaching the singular limit $\epsilon\to0$, both boundary values change. Therefore, we also continued $g_{M}^{*}$ in parameters *ϵ* and *J*, to obtain the *J* value at the boundary at the singular limit. Figure 15B indicates that at the singular limit $\epsilon\to0$, the boundary value for *J* is slightly less than 0.2. Note that the parameters mentioned from now on do not have units, because we are considering the singular limit of a dimensionless system.

To approximate the singular orbit of (10) in Fig. 8, we use $\epsilon=0.001$ in the simulations. According to Fig. 15, we need another set of $g_{M}$, *J*, and $I_{\mathit {ton}}$ values to simulate the phase-locking and non-phase-locking scenarios. In Fig. 8B, we control the input frequency by fixing $J=0.16$. The two levels of $g_{M}$ we chose are −0.3 and −0.5. In order to ensure that the natural frequencies of the I-cell are the same at these two levels of $g_{M}$ and $\epsilon=0.001$, we set $I_{\mathit {ton}}=1$ when $g_{M}=-0.5$ and $I_{\mathit {ton}}=5$ when $g_{M}=-0.3$. Using these parameters, the I-cell phase-locks to the E-cell (with $J=0.16$) at the high M-current level ($g_{M}=-0.3$, $I_{\mathit {ton}}=5$), but does not phase-lock to the E-cell at the low M-current level ($g_{M}=-0.5$, $I_{\mathit {ton}}=1$).

## Appendix 5: Choice of Fixed *w* in Fig. 8

To project the folds (16) in $(v,v_{2},s)$ space, we need to fix a *w* value in addition to the approximations we made in (18). Since we want to know what contributes to the timing of the spiking of the I-cell, we chose a typical *w* value before the I-cell spikes. Figure 16 shows two simulations of the approximated singular orbit ($\epsilon=0.001$) with two levels of maximal M-current conductance. With low $g_{M}$ (Fig. 16A), *w* is around 0.74 before spiking. With high $g_{M}$ (Fig. 16B), *w* is around 0.7 before spiking. Hence, in Fig. 8B, we fix $w=0.74$ for $g_{M}=-0.5$ (green I-fold) and $w=0.7$ for $g_{M}=-0.3$ (red I-fold).

## Appendix 6: Equations of the h-Current

The equations of the h-current we use in the simulations are as follows. They are the same as in [37].

23 $$\begin{aligned} I_{h}&=2(0.35mhs+0.65mhf) (-20-v), \\ mhs'&=\frac{(mhs_{\infty}(v)-mhs)}{\tau_{mhs}(v)}, \\ mhf'&=\frac{(mhf_{\infty}(v)-mhf)}{\tau_{mhf}(v)}, \\ mhs_{\infty}(v)&=\frac{1}{1+e^{(v+71.3)/7.9}}, \\ mhf_{\infty}(v)&=\frac{1}{1+e^{(v+79.2)/9.78}}, \\ \tau_{mhs}(v)&=\frac{5.6}{e^{(v-1.7)/14}+e^{-(v+260)/43}}+1, \\ \tau_{mhf}(v)&=\frac{0.51}{e^{(v-1.7)/10}+e^{-(v+340)/52}}+1. \end{aligned}$$

## Abbreviations

CFC: cross-frequency coupling
E-cell: excitatory cell
GABA: gamma-aminobutyric acid
I-cell: oscillator with an inhibitory autapse
PING: pyramidal-interneuronal network gamma
SNIC: saddle node on invariant circle
STDP: spike-time-dependent plasticity
